# Impact of Volatile Organic Compounds on the Growth of *Aspergillus flavus* and Related Aflatoxin B1 Production: A Review

**DOI:** 10.3390/ijms232415557

**Published:** 2022-12-08

**Authors:** Laurie Josselin, Caroline De Clerck, Marthe De Boevre, Antonio Moretti, Marie-Laure Fauconnier

**Affiliations:** 1 Laboratory of Chemistry of Natural Molecules, Gembloux Agro-Bio Tech, Liege University, Passage des déportés 2, 5030 Gembloux, Belgium; 2 AgricultureIsLife, Gembloux Agro-Bio Tech, Liege University, Passage des Déportés 2, 5030 Gembloux, Belgium; 3 Centre of Excellence in Mycotoxicology and Public Health, Department of Bioanalysis, Faculty of Pharmaceutical Sciences, Ghent University, Ottergemsesteenweg 460, 9000 Gent, Belgium; 4 Institute of Sciences of Food Production, National Research Council of Italy, Via Amendola 122/O, 70126 Bari, Italy

**Keywords:** volatolome, fungal growth, Aflatoxin B1 control, bioactive volatile organic compounds

## Abstract

Volatile organic compounds (VOCs) are secondary metabolites of varied chemical nature that are emitted by living beings and participate in their interactions. In addition, some VOCs called bioactive VOCs cause changes in the metabolism of other living species that share the same environment. In recent years, knowledge on VOCs emitted by *Aspergillus flavus*, the main species producing aflatoxin B1 (AFB1), a highly harmful mycotoxin, has increased. This review presents an overview of all VOCs identified as a result of *A. flavus* toxigenic (AFB1-producing) and non-toxigenic (non AFB1-producing) strains growth on different substrates, and the factors influencing their emissions. We also included all bioactive VOCs, mixes of VOCs or volatolomes of microbial species that impact *A. flavus* growth and/or related AFB1 production. The modes of action of VOCs impacting the fungus development are presented. Finally, the potential applications of VOCs as biocontrol agents in the context of mycotoxin control are discussed.

## 1. Introduction

Recently, volatile organic compounds (VOCs), which are categorized as secondary metabolites, have risen to general attention and been widely studied. VOCs are known to actively participate in inter- and intra- living species communication [1,2,3]. In particular, VOCs are becoming the new frontier in the metabolomics field. With the development of new technologies, the fields of application of VOCs, such as the biomedical field, have grown over the last few years. In addition, VOCs have been investigated in-depth for the roles they also play in soil, in influencing atmospheric chemistry, and in microbe–microbe, plant–microbe, and plant–plant interactions [4].

Much research has also addressed the influence of certain VOCs or volatolomes (the set of VOCs emitted by a given species) on fungi by observing their antifungal, antibiotic, antimicrobial properties and others [5].

Interestingly, these secondary metabolites oftentimes share the same biosynthetic pathway as some mycotoxins [6]. Thus, much work has been also devoted to analyzing the impact of the VOCs on inhibiting the ability of some mycotoxigenic fungi to produce mycotoxins. In particular, VOCs from several sources have been shown to inhibit the production of Ochratoxin A by *Aspergillus carbonarious* [4] and aflatoxins by *Aspergillus flavus* [7].

*Aspergillus flavus* is a fungal species that causes serious damage to crops in the field or during storage [8]. Beyond its pathogenic effects on several crops, this fungus is also the main producer of the mycotoxin aflatoxin B1 (AFB1) [9]. This mycotoxin contributes to major health problems worldwide as well at sanitary and economic levels [10,11]. AFB1 remains active even after heat treatments used in conventional food manufactures since it is a thermostable compound [12]. Chronic exposure towards AFB1 leads to multiple diseases such as hepatocellular cancer, and acute consumption beyond the maximum permissible limits can lead to the death of the individual [13,14,15]. This makes AFB1, to date, a serious threat to humans, but also the most controlled mycotoxin via European and worldwide legislation [16]. In recent years, the methods of detection have become increasingly powerful and sensitive, and accurate techniques promoting an easier detection have been developed [17,18]. At the same time, the concern and the need to control the contamination and the production of AFB1 in order to mitigate its occurrence in crops have emerged [19].

In particular, some VOCs identified here as bioactive VOCs have promising ability to affect both *A. flavus* growth and AFB1 production. Among the works compiled, we can distinguish among those focused on the evaluation of mycelial growth, other works investigating the effects on AFB1 production, and studies aiming to elucidate the impact of VOCs on the gene expression of the aflatoxin biosynthetic pathway gene cluster.

Among the tools to reduce the impact of mycotoxins on crops, the early detection of both fungi and mycotoxins is a key approach. Such detection can be achieved by a range of different markers, such as DNA-based markers for the fungi, or rapid kits for easy and fast chemical analyses of mycotoxins [20,21]. A recent trend aims to develop species-specific markers based on specific VOC profiles emitted by fungi. The monitoring of the emission of VOCs over several days of growth of *A. flavus* has revealed many VOCs, some of which are commonly emitted also by other fungal or other microbial species, whereas others are considered specific to this species.

*A. flavus* includes two kinds of strains based on their ability to produce AFB1. The biosynthesis of AFB1 is linked to the presence of a cluster consisting of 30 genes (*afl*) on chromosome 3 [22]. The toxigenic strains (TS) possess the entire gene cluster involved in aflatoxin biosynthesis that gives to strains the ability to produce AFB1, whereas non-toxigenic strains (NTS) lack some of these genes [23]. In addition, NTS are not genetically identical since they can lack different number and kind of *afl* genes [24]. Finally, although TS and NTS share the same environment, they are genetically incompatible and there are no examples of hybridization between them [25].

NTS and TS of *A. flavus* share the same environment and can be both frequently isolated from same parts of plants or soils. Therefore, it is important to correctly identify TS and NTS to accurately evaluate the risk related to *A. flavus* occurrence. Molecular markers are not available for *A. flavus* since, as mentioned above, several genetic patterns of NTS can occur. On the other hand, chemical analyses, even using rapid kits, can require much time and laboratory resources. Therefore, the identification of specific VOCs for NTS and/or TS strains of *A. flavus* could provide further markers for an early and reliable assessment of strain toxigenicity.

This review will address four main questions related to *A. flavus*, VOCs and AFB1:

- Which VOCs are emitted by *A. flavus* and are specific to TS or NTS?
- Which bioactive VOCs or volatolomes of various origins affect the growth of *A. flavus* and/or its production of AFB1?
- What are the modes of action of these bioactive VOCs?
- How can we exploit these VOCs to our advantage to control the growth of *A. flavus* and its AFB1 production?

## 2. Which VOCs Are Produced by *A. flavus* and Are Specific to TS or NTS?

### 2.1. The Diversity of VOCs Emitted by A. flavus

VOCs include a wide range of molecules (alcohols, esters, furans, ketones, aldehydes, terpenes, hydrocarbons, i.a.) with low molecular weight and high vapor pressure. These VOCs are emitted by many natural and anthropogenic sources. Concerning natural VOCs, different terminologies are used depending on their origin. Biogenic volatile organic compounds (bVOCs) are defined as the volatile compounds that are emitted by living beings [26,27]; the VOCs emitted by microorganisms (including fungi and bacteria) can be referred to as microbial volatile organic compounds (mVOCs) [28]. Finally, in a more specific way, the VOCs produced by fungi are defined as fungal volatile organic compounds (fVOCs) [29].

VOCs are emitted by fungi in order to fulfill different internal or external functions for the fungus [3]. The emission of some VOCs can inhibit certain functions of the fungus or fungal structures [30]. Thus, germination, mycelium growth, and sporulation can be regulated by the emission of VOCs. Other VOCs are involved in interactions with other kinds of living organisms. Some VOCs attract insects to maximize fungal dissemination [1], some interact with the host plants to weaken their defenses [31], and other VOCs have antimicrobial activity and thus limit the colonization of other fungal or microbial species that may compete for the substrate, or even control the population of the microorganism that produces them, a phenomenon called quorum sensing [32,33].

Almost 400 VOCs emitted by *A. flavus* have been reported in the literature so far, as identified from the volatolomes emitted by the various strains analyzed. A synthesis of these VOCs (listed as a whole in the Table S1) is presented in Table 1. This table illustrates their great diversity from a chemical class standpoint. Table 1 also presents the total number of VOCs emitted for each chemical family and whether these VOCs are emitted more specifically by TS or NTS of *A. flavus*. The strains for which we lack the information on their toxigenicity are reported in the Table as unknown (US). Many studies examined both a TS and a NTS and thus compared their volatolomes.

More than 50 different compounds belonging to four chemical families (alcohol, alkane, alkene and terpene) have been reported. The alcohol class includes the highest number of identified VOCs (3-methylbutan-1-ol, ethanol), as well as those associated with the typical odor of the fungi (oct-1-en-3-ol, octan-3-ol) [34,39]. In the alkane class, there is a predominance of compounds ranging from 5 to 19 carbons, while only three compounds containing more than 30 carbons and 16 cyclic structures have been listed. Within the family of alkenes, aromatic and cyclic compounds such as derivatives of xylene or styrene were often found (up to 40% of the total). The terpene group is composed of monoterpenes and sesquiterpenes, with a great predominance of the latter.

Some recurrent VOCs are always detected as emitted by *A. flavus* strains, such as 3-methylbutan-1-ol, 2-methylpropan-1-ol, ethyl acetate and 2-methylfuran, making them potential markers of *A. flavus* occurrence.

In Table S1 some trends associated with the toxigenicity of *A. flavus* strains are highlighted. From a general point of view, TS emit a greater diversity of chemical families than NTS. Indeed, all chemical families are emitted and are widely represented, especially terpenes with more than 40 specific VOCs, followed by ketones and hydrocarbons (alkane and alkene). Only a single monoterpene emitted exclusively by NTS has been identified: p-mentha-1,3,8-triene. To our knowledge and to date, some VOCs are assimilated as specific to a category of *A. flavus*, as it is the case of pent-2-yn-1-ol for TS [35]. Other VOCs, such as epi-bicyclosequiphellandrene, 2-phenoxyethanol or γ-gurjunene, are supposed to be specific to TS but due to the lack of information about the studied strains, their exclusivity to this category cannot be fully confirmed.

It is necessary to underline that the specificity of some VOCs for NTS vs TS and vice-versa does not exclude the possibility that some of them are produced by other fungal or microbial species.

### 2.2. VOCs Emission of A. flavus Influenced by Biotic and Abiotic Factors

A significant variability in the number and amounts of VOCs emitted by *A. flavus* and in its growth kinetics has been reported. Sun et al. (2014) showed that the VOCs emitted by a NTS were more abundant than those emitted by a TS [39]. Josselin et al. (2021) have observed the opposite trend that TS can emit larger amounts of VOCs compared with a NTS, with the majority of these VOCs belonging to the terpene family. This latter study also highlighted a change in the volatolome of a natural mutant unable to produce AFB1, obtained from a TS. For this mutant strain, in addition to its loss of AFB1 production, a concurrent difference in the emission of certain terpenes was observed [36]. In conclusion, the nature of the strain itself brings variability to the volatolome released by *A. flavus*.

The effects of an increase in temperature on VOC emissions in TS of *A. flavus* was also studied by Sun et al. (2014) and showed fluctuations in terpene and alcohol contents (ethanol, butan-1-ol, 3-methylbutan-1-ol and 2-methylbutan-1-ol). For example, a temperature higher than 37 °C seems to inhibit the production of terpenes, although they were abundant during the analyses carried out at 30 °C and 15 °C [40]. Growth temperature is thus an important parameter when considering VOC emission by *A. flavus*. On the other hand, water activity and pH are also frequently mentioned as parameters that influence fungal growth and AFB1 production [47,48,49]. However, data that relate these parameters and studies on VOCs are lacking. The growth media also influences VOC emission, as reported by De Lucca et al. [34,35], who pointed out that maize media resulted in a greater number of VOCs compared with PDA medium. In addition, Sun et al. (2016) showed that the number of terpenes emitted increased if the carbon source was more accessible [40].

The method of VOC sampling can also influence the VOCs detected. Among the methods, the most common static method used is the SPME, which leads to adsorbing a large range of chemical families, while the dynamic head space method used is most often performed with a TENAX tube for the same reason. The importance of the SPME parameters was highlighted by Sun et al. (2016) by comparing the number, the amount and the chemical families sampled [40].

## 3. Which Bioactive VOCs or Volatolome of Various Origins Affect the Growth of *A. flavus* and/or Its Production of AFB1?

In order to examine all the bioactive VOCs leading to a modification of the growth of *A. flavus* and/or its production of AFB1, the VOCs were grouped according to their origin of emission. Thus, the volatolomes of microorganisms such as fungi, bacteria or yeasts, the VOCs from plant extracts such as essential oils and, finally, the individual and pure VOCs are detailed in the three sections below.

**Figure 1:**
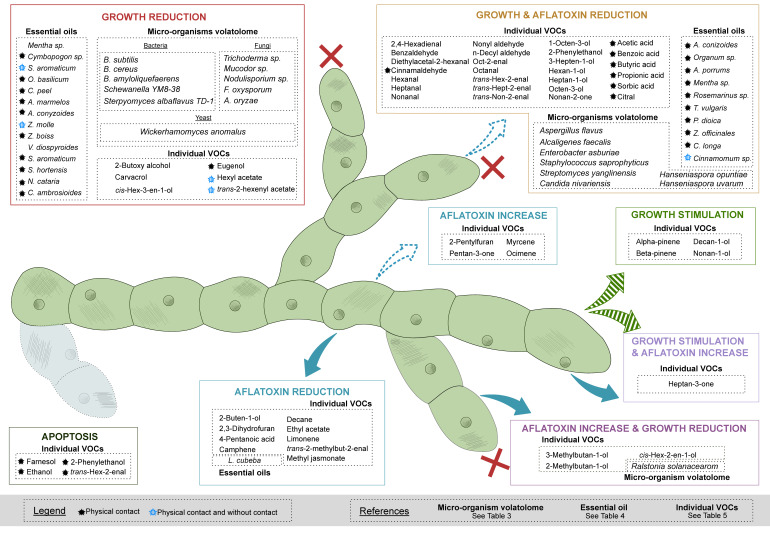
Summary of bioactive VOCs and species volatolomes influencing growth and AFB1 production parameters of *A. flavus*.

Figure 1 shows all the studied volatolomes or bioactive VOCs in eight categories, listing the changes observed in the two targeted parameters (growth and AFB1 production). The majority of the compounds are active mainly on fungal growth, and some also act on AFB1 production. In contrast, the above mention parameters can be stimulated by some given bioactive VOCs or volatolomes, as reported by Cleveland et al. (2009) and Zeringue et al. (1990) [50,51].

The main patterns in the effects of volatolomes, essential oils and individual VOCs on *A. flavus* growth and related AFB1 production are summarized in Table 2. According to the information known today, it seems that some families of compounds such as alcohols or terpenes can cause either inhibitory effects on the growth and the production of AFB1 or stimulate them, although the prevailing tendency of the studied bioactive VOCs or volatolomes is a reduction in the above-mentioned parameters. However, the trends observed for *A. flavus* are not always identical to those found when a wider species or genus of fungi are considered, demonstrating that each species reacts differently to the same bioactive VOCs. This is notably the case for aldehydes that are extremely efficient at reducing *A. flavus* growth and turn out to have an effect of increasing fungal growth of other species such as *F. oxysporum, Colletotrichium fragarie* or *Botrytis cinerea* [52]. Table 2 also shows that the mode of application of the individual VOCs also influences the effects on the two parameters, which will be discussed in the section devoted to them.

### 3.1. The Volatolomes from Bacteria, Yeast and Fungi Reduce the Growth of A. flavus

The effects on growth and AFB1 production caused by panels of volatolomes released by microorganisms (bacteria, yeast and fungi) on *A. flavus*, without physical contact between *A. flavus* colonies and the emitting species, are reported in Table 3. In general, the fungistatic effect is often the primary parameter to be studied; therefore, data regarding the effect on AFB1 production are sometimes not available. In the case of fungi, bioactive VOCs can impact a wide range of parameters, including sporulation, conidia germination and different morphological modifications of their living structures (e.g., hyphae) [53,54].

The VOC-producing species most frequently investigated for their effects against *A. flavus* belong to the *Muscodor* and *Trichoderma* fungal genera, and *Streptomyces* and *Bacillus* bacterial genera [55,56]. Following a screening of 75 *Bacillus* strains, significant reductions in the growth of *A. flavus* in the presence of *Bacillus subtilis, B. cereus* and *B. amyloliquefaciens* volatolomes were noted [57]. In addition, the volatolomes of the above-mentioned *Bacillus* species had a significant impact also on other toxigenic fungi, such as *Aspergillus niger*, *Fusarium graminearum, F. oxysporum* and *F. verticillioides*, on other fungal pathogens, and even on other organisms such as nematodes.

The fungal species reported in Table 3 are all endophytic fungi [58]. Concerning endophytic fungi, the experiments were carried out by physically separating the strains in order to evaluate only the action of VOCs, which is indeed a little different from the conditions found in the plant where non-volatile compounds could also play a role. The most abundant VOC produced by *A. oryzae*, 1-octen 3 ol, was found to increase AFB1 production with a dose-dependent effect. Moreover, *A. faecalis* [59], *E. asburiae* [60], *Staphylococcus saprophyticus* [61] and *A. flavus* itself [62] produced VOCs that induced a reduction in AFB1 production, whereas VOCs of *Ralstonia solanacearum* stimulated its production.

Bacterial and fungal volatolomes can also affect other developmental parameters of *A. flavus*. Several effects were reported by Gong et al. (2020) and Braun et al. (2012) including inhibition of pectin methylesterase, cellulase and polyphenol oxidase secretion, conidial germination, sexual development and cell damage [61,63]. Interestingly, the effects are reciprocal, as was the case with *Ralstonia solanacearum* where a reduction in the growth of the bacterium and its melanin production was observed, probably induced by an increase in AFB1 production by *A. flavus* [62]. A characterization of the volatolomes of some species has been performed, making it possible to relate the effects observed on *A. flavus* and the VOCs with bioactive potential [55,59,60,63,64,65,66].

**Table 3:** Volatolomes and their major compounds when identified from bacteria, yeast and fungi impacting *A. flavus* growth and/or its AFB1 production without physical contact.

Species and Main VOCs		Impact		References	
		Growth	Aflatoxin		
**Bacteria**	***Alcaligenes faecalis*** Dimethyl disulfide Methyl 3-methylbutanoate	-	-	Gong et al., 2019	[59]
	***Bacillus subtilis***	-	NA	Chaves-López et al., 2015	[57]
	***Bacillus cereus***	-	NA	Chaves-López et al., 2015	[57]
	***Bacillus amyloliquefaciens***	-	NA	Chaves-López et al., 2015	[57]
	***Enterobacter asburiae*** 1-Methoxy-3-methylbutane Pentan-1-ol 2-Phenylethanol	-	-	Gong et al., 2019	[60]
	***Ralstonia Solanacearum***	-	+	Spraker et al., 2014 Singh et al., 2020 Suwannarach et al., 2013	[44] [55] [64]
	***Schewanella* algae** Dodecan-2-ol 2,4-bis(1,1-Dimethylethyl)-phenol 2,2-Dimethyl-oxazole Butylated hydoxytoluene Nonane Dimethyl trisulfide	-		Gong et al., 2015	[66]
	***Staphylococcus saprophyticus*** 3,3-dimethyl-1,2-epoxybutane	-	-	Gong et al., 2020	[61]
	***Streptomyces philanthi***	-		Boukaew and Prasertsan, 2020	[56]
	***Streptomyces yanglinensis***	-	-	Lyu et al., 2020	[67]
**Yeast**	***Candida nivariensis*** 2-Methylpropan-1-ol 3-Methylbutan-1-ol Pentan-1-ol	-	-	Jaibangyang et al., 2020	[68]
	***Hanseniaspora opuntiae*** Acetic acid 2-Methylbutanoic acid 2-Phenylethyl acetate	-	-	Tejero et al., 2021	[69]
	***Hanseniaspora uvarum*** Ethyl acetate 3-Methylbutan-1-ol 2-Methylbutan-1-ol 2-Phenylethyl acetate	-	-	Tejero et al., 2021	[69]
	***Wickerhamomyces anomalus*** 2-Phenylethanol	-	NA	Tilocca et al., 2020	[65]
**Fungi**	***Streptomyces alboflavus***	-	NA	Yang et al., 2019	[70]
	***Fusarium oxysporum*** Limonene	-	NA	Suwannarach et al., 2013	[64]
	***Muscodor genus*** 2-Methylpropanoic acid 2- Methylbutan-1-ol 3-Methylbutan-1-ol	-	NA	Braun et al., 2012 Singh et al., 2020 Suwannarach et al., 2013	[63] [55] [64]
	***Nodulisporium sp.*** 1,8 Cineole Terpinen-4-ol	-	NA	Suwannarach et al., 2013	[64]
	***Trichomderma genus***	-	NA	Singh et al., 2020	[55]
	***Aspergillus flavus***	-	-	Sweany and Damann, 2020	[62]
	***Aspergillus oryzae*** Octa-1,3-diene Octa-1,5-diene-3-ol 1-Octene-3-ol Octan-3-one Octanal Oct-2-enal 1-Octene-1-ol Octa-2,4-dieneal	-	NA	Singh et al., 2020	[55]

(+) Increase, (-) Reduction, (NA) data not available.

### 3.2. Blends of VOCs from Essential Oils Show Antifungal Properties and Regulation Effects on AFB1 Production in A. flavus

For many years, essential oils have been the subject of numerous studies on their properties, including their efficiency as antifungals. With regard to *A*. *flavus*, the efficiency of this property has been by using two modalities: (i) during a contact between *A. flavus* and the essential oil (by using discs or by introducing it directly into the culture medium), or (ii) without direct physical contact between the fungus and the essential oil (by fumigation or by introducing a volume of essential oil in a closed space containing the colony of *A. flavus*) (Table 4). An essential oil is a mixture of VOCs, often consisting of mono- and sesquiterpene, benzoids and other classes of molecules, resulting from the natural extraction from a plant. Many terpenes discovered in recent decades that are components of essential oils, have various associated activities such as anti-phytopathogenic, immunosuppressive, anti-inflammatory, anti-bacterial, cytotoxic, antifungal, anti-viral activities as well as enzyme inhibition, among others [71].

All the tested essential oils produced a fungistatic effect regardless the mode of application (contact or not) with the fungus, with the exception of *Litsea cubeba*, although this essential oil produced an inhibition of AFB1 production. The essential oils in the Table 4 are non-specific to *A. flavus* and also affect other fungal species, including those belonging to the *Aspergillus* genus.

Two opposing approaches have been tested. On the one hand, the complexification of the mixtures to improve the synergy of the constituent molecules of the essential oils has been investigated. *Cinnamomum, Origanum* and *Thymus*, taken individually, have been shown to have a significant impact. However, it is the combination of the three that induced a much more effective synergy, causing a down-regulation of aflatoxin biosynthesis genes (70% inhibition of aflatoxins) and an associated decrease in the total growth of *A. flavus* colonies [72,73,74]. On the other hand, the simplification of mixtures by determining the VOCs associated with antifungal and anti-aflatoxigenic effects, starting with their major compounds, has been studied. In this case, the antifungal effect of the essential oil of *Cinnamomum cassia* was compared with its main compound, cinnamaldehyde. Both showed inhibition of the development of *A. flavus* and *A. oryzae*, but according to the data presented, the single molecule was more effective than the whole essential oil [73].

Some of the *A. flavus* antagonistic molecules emitted by the microorganisms listed in Table 3 are also present in the essential oils listed in Table 4. This is the case for 1,8-cineole and limonene, the latter of which appears as a constituent of six of the essential oils observed here.

**Table 4:** Essential oils and their major VOCs impacting the growth of *A. flavus* and/or its production of AFB1.

(a) Latin Name and Major VOCs	(b) Impact		(c) Application Mode	References	
	Growth	Aflatoxin			
***Aegle marmelos*** D and L-Limonene *	-	NA	Contact	Adorjan and Buchbauer, 2010	[75]
***Ageratum conyzoides*** Precocene I and II Dimetoxy ageratocromene Ageratocromene	-	-	Contact	Adorjan and Buchbauer, 2010 Esper et al., 2014	[75] [72]
***Allium porrums*** Diallyl trisulfide Diallyl disulfide Methyl allyl trisulfide 5-Ethylthiazole	-	-	Contact	Kocevski et al., 2013 Abd El-Aziz et al., 2015	[73] [76]
***Capsicum*** Not available	-	NA	No contact	Boukaew et al., 2017	[77]
***Chenopodium ambrosioides*** (Z)-Ascaridole	-	NA	Contact	Adorjan and Buchbauer, 2010	[75]
***Cinnamomum*** Cinnamaldehyde (E)-2-methoxycinnamaldehyde Carveol α-Cadinol	-	-	Contact No contact	Abd El-Aziz et al., 2015 Boukaew et al., 2017 Manso et al., 2013 Kocevski et al., 2013 Xiang et al., 2020	[76] [77] [78] [73] [74]
***Citrus peel*** Limonene * Linalool Citral	-	NA	Contact	Taguchi et al., 2015	[79]
***Curcuma longa L.*** Ar-Tumerone α –Tumerone β-Tumerone Ar-Curcumene β -Sesquiphellandrene	-	-	Contact	Ferreira et al., 2013 Hu et al., 2017	[80] [81]
***Cymbopogon*** (Z)-Citral (E)-Citral Limonene *	-	NA	Contact	Xiang et al., 2020	[74]
***Litsea cubeba essential*** (Z) and (E)-Limonene oxide D-Limonene *	NA	-	Contact No contact	Li et al., 2016	[82]
***Mentha*** Menthol Menthone Menthyl acetate Menthofurane	-	-	Contact	Abd El-Aziz et al., 2015 Beyki et al., 2014 Taguchi et al., 2015	[76] [83] [79]
***Nepeta cataria*** 4aa,7a,7ab-Nepetalactone	-	NA	Contact	Adorjan and Buchbauer, 2010	[75]
***Ocimum basilicum*** Linalool Methylchalvicol Eugenol Methyl eugenol Methyl cinnamate 1,8- Cineole Caryophyllene *	-	NA	Contact	Taguchi et al., 2015 Xiang et al., 2020	[79] [74]
***Origanum*** Carvacrol Thymol 4-Terpineol Linalool γ-Terpinene α-Terpineol	-	-	Contact	Esper et al., 2014 Xiang et al., 2020	[72] [74]
***Pimenta dioica*** α-Terpinoel β-Linalool γ-Terpinene Eucalyptol	-	-	Contact	Kumar Chaudhari et al., 2022	[84]
***Pogostemon cablin*** Patchouli alcohol 4-Oxo-14-norvitrane δ-Guaiene	-	NA	Contact	Kocevski et al., 2013	[73]
***Rosemary*** Camphor 1,8-Cineole α-Pinene * Verbenone Camphene Limonene * Bornyl acetate α-Terpineol β-Pinene	-	-	Contact	Abd El-Aziz et al., 2015 Taguchi et al., 2015	[76] [79]
***Satureja hortensis*** Thymol Carvacrol	-	NA	Contact	Adorjan and Buchbauer, 2010	[75]
***Syzygium aromaticum*** Eugenol Eugenyl acetate Caryophyllene Benzenemethanol	-	NA	Contact No contact	Adorjan and Buchbauer, 2010 Boukaew et al., 2017 Taguchi et al., 2015 Xiang et al., 2020	[75] [77] [79] [74]
***Thymus vulgaris*** p-Cymene γ-Terpinene Thymol	-	-	Contact	Abd El-Aziz et al., 2015 Khalili et al., 2015	[76] [85]
***Vatica diospyroides*** Symington Benzyl acetate Benzyl benzoate Isoeugenol α-Terpineol	-	NA	No contact	Boukaew et al., 2017	[77]
***Zanthoxylum molle*** Undecan-2-one Limonene * Terpinen-4-ol	-	NA	Contact No contact	Tian et al., 2014	[86]
***Zataria multiflora Boiss*** Carvacrol	-	NA	Contact	Adorjan and Buchbauer, 2010	[75]
***Zingiber officinale*** β-Phellandrene Zingiberene Geranial Neral	-	-	Contact	Adorjan and Buchbauer, 2010 Nerilo et al., 2016 Taguchi et al., 2015	[75] [87] [79]

(a) Latin name of the plant and majority VOCs identified in essential oils. When a VOC constituting an essential oil is known to be emitted by *A. flavus* species (in accordance with the Table S1) it is indicated by an asterisk * in column (a). (b) Compilation of (-) inhibitory or (NA) unavailable data for growth and production of AFB1. (c) Contact type (contact/non-contact).

### 3.3. Single Bioactive VOCs Affecting the Growth and/or the AFB1 Production of A. flavus

The individual bioactive VOCs are produced by fungal species, microorganisms and plants, but to our knowledge, no study on the influence of the complete plant volatolome on *A. flavus* or mycotoxin production has been undertaken. Among the 64 individual bioactive VOCs affecting the growth of *A. flavus* and/or its production of AFB1, there are 27 VOCs known to be emitted by the species *A. flavus* itself (Table 5). Within these bioactive VOCs, we find nonan-2-one and octan-3-one [50] or trans-2-methylbut-2-enal and 2,3-dihydrofuran [7] specifically emitted by NTS, or decan-1-ol and limonene [50] emitted by TS.

Four molecules with fungicidal action resulting in cell death have been reported. All of them were studied following physical contact with colonies of *A. flavus*. These studies showed that hexanal (0.4 µL/mL) [99], 2-phenylethanol (lethal at 0.3–0.5%) [89], farnesol (400 µM) [102] and nonan-1-ol (20 µL/mL) [54] lead to fungal death due to the loss of its membrane integrity.

All other individual bioactive VOCs have fungistatic effects toward *A. flavus* associated with variable AFB1 production responses. A reduction in the mycelial structure does not necessarily extend to the other fungal structures, as is the case for trans-hex-2-enal (diluted in ethanol) which causes lethality to the mycelia (95% at 20 µM) but does not affect conidia viability [100].

Total inhibition of AFB1 and fungal growth was observed with benzaldehyde, hexanal, nonyl aldehyde, trans-non-2-enal, heptanal and octanal by using different concentrations in a contactless approach [50,51,92]. In particular, Cleveland et al. (2009) showed a significant influence on the AFB1 production of the VOCs concentration used, highlighting that the mechanisms leading to AFB1 production are more sensitive than those involved in growth reduction [51]. Additionally, the inhibition of spore germination with trans-hex-2-enal, hexanal, trans-non-2-enal and 2-methylpropionic acid was observed and further damage by their hydroperoxide metabolites via lysis of hydroperoxides was also noted [63,103]. A positive correlation was established between AFB1 and the amount of 1-octen-3-ol, although this compound induced a reduction in *A. flavus* growth, sclerotia and conidia density [55]. In addition, an increase in alpha-amylase production by *A. flavus* was also observed as a consequence of 1-octen-3-ol presence [55].

Furthermore, it has been proved that each molecule has its own minimum concentration that affects the growth of *A. flavus* colonies and/or AFB1 production, and this concentration can be highly variable [51,91]. In addition, for each molecule, the frequency of exposure (punctual or cyclic) is also important [100,103].

Even if no changes are observed in the growth of the mycelium of *A. flavus*, other effects may be observed in the colonies, such as suppression of spore germination, changes in mycelial pigments (notably, observed for methyl jasmonate) [103], and reduction of AFB1 [50]. Modifications due to the substrates on which *A. flavus* was grown were also observed. The growth inhibition when *A. flavus* was grown on maize seeds or PDA medium are similar, but differences concerning AFB1 production were observed [72,76].

Some VOCs can exacerbate the production of AFB1. 3-Methylbutan-1-ol, 2-methylbutan-1-ol, cis-hex-2-en-1-ol, myrcene, ocimene, 2-pentylfuran and hexan-3-one did not affect *A. flavus* growth, but increased AFB1 production up to 50%, with a higher trend for the first two mentioned alcohols [50,51]. In particular, 3-methylbutan-1-ol and 2-methylbutan-1-ol are mainly emitted by fungal species that are competitors of *A. flavus*. Thus, one hypothesis is that their presence could stimulate the “defense system” of *A. flavus*, leading to the synthesis of AFB1.

Only a fungistatic effect for *A. flavus* has been reported on vaporization of decan-1-ol, alpha and beta-pinene [50] or with 2-butoxy alcohol [50] and furfural [98]. However, it is interesting to note that, three of these compounds are naturally emitted by the same *A. flavus*, specifically, decan-1-ol, furfural and alpha-pinene.

The expression of divergent effects triggered by the same VOC has also been underlined by Zhang et al. (2021). They found that the growth of *A. flavus* showed a negative correlation with an increasing concentration of sprayed nonan-1-ol [54]; however, the opposite trend was detected by Zeringue et al. (1990)*,* who showed that vaporization increased the mycelium growth, in addition to creating oxidative stress in the mycelium [50].

A comparison between fumigation and contact mode reveals that experiments carried out using fumigation required lower concentrations than those performed using contact, with respect to mycelium inhibition. Ma et al. (2017) determined that a fumigation with a 50-fold lower concentration of trans-hex-2-enal than the concentration used by physical contact was required for growth inhibition of *A. flavus* [90], and the same trend was noted with the essential oils [77,82,86].

## 4. What Are the Modes of Action of These Bioactive VOCs?

**Figure 2:**
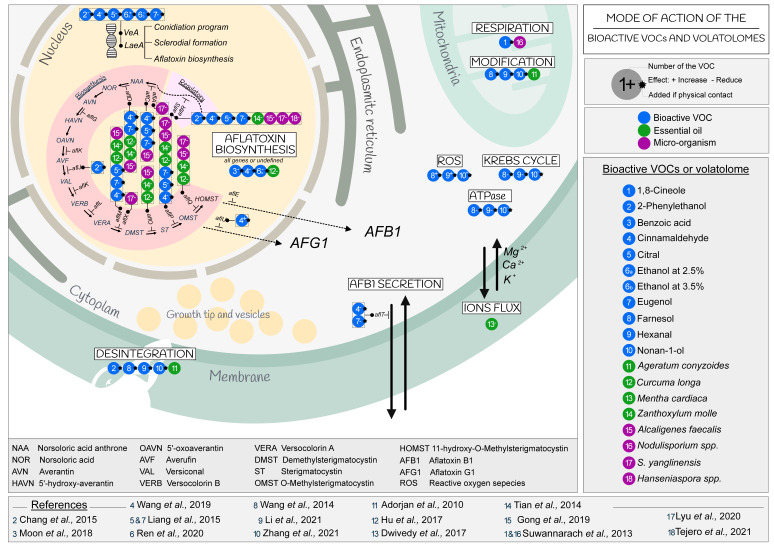
Mode of action of the bioactive VOCs and volatolomes on *A. flavus* [53,54,59,64,67,69,75,81,86,88,91,94,96,99,102,104].

Although fungicidal, fungistatic, or AFB1-reducing effects induced by several bioactive VOCs or volatolomes have been proved, few of these have been further investigated for the mechanisms that are involved in such activities. Regarding the AFB1 production, some studies have focused on the gene expression of some selected *afl* genes. In addition, the impact of VOCs on mycelial growth, sporulation and the germination of conidia or on physiological functions and genetic mechanisms have been rarely studied. To date, investigations have focused on mechanisms such as the loss of fungal membrane integrity and the regulation of the AFB1 biosynthetic gene cluster (Figure 2).

### 4.1. Loss of Membrane Integrity of A. flavus

The loss of membrane integrity is the result of several forms of deregulation of the physiological functions of *A. flavus*. A systematic observation of the endomembrane system, mainly of the plasma membrane and mitochondria, of *A. flavus* cells rapidly detected the induction of structural changes after exposure to some VOCs. 2-Phenylethanol, farnesol, hexanal, nonan-1-ol and *Ageratum conyzoides* essential oil caused shrinkage and detachment of the cell wall in the cytoplasm. An alteration of the mitochondria membrane, which became less defined and discontinuous or absent, was observed due to changes in their lipid and fatty acid composition, in addition to the down regulation of the mitochondrial dehydrogenases [54,88,89,99,102,105]. On the other hand, essential oils (*Zanthoxylum molle, Ageratum conyzoides*) that are mixtures of several compounds could also disrupt all membranes by crossing the layers of polysaccharides, fatty acids and phospholipids, changing the pH, and dramatically modifying the physiological functions of the cell [75,86]. According to Basak et al. (2018), the main mode of action of essential oils was related to the permeability of organelles [106]. A further impact of *Mentha cardiaca* essential oil on *A. flavus* was the leaking of Ca^2+^, K^+^ and Mg^2+^ ions from cell membranes, as indicated by measurements of the electrical conductivity [54,99,102,104]. This caused accumulation of ROS (reactive oxygen species), disruption of the Krebs cycle (or TCA) and reduction of ATPase [54,102]. Considered together, the effects of essential oils show an enormous capability to alter several cellular functions in *A. flavus* and thereby affect its fitness and survival possibilities.

### 4.2. Modification of Afl Gene Expression

The studies on AFB1 biosynthesis gene expression are not all focused on the same genes and are not exhaustive.

Some studies proved that 2-phenylethanol, cinnamaldehyde, citral, eugenol and ethanol act on global regulatory genes such as the velvet complex (*VeA*) or the *LaeA* gene in *A. flavus* [53,94,96]. In addition to the aflatoxin biosynthetic pathway, these regulatory genes are also involved in the regulation of sexual development, sclerotia formation, and conidia programming [22].

Interestingly, the concentration of a single applied molecule can differentially affect gene expression. While 2.5% ethanol increased the regulation of the velvet complex, a concentration of 3.5% of the same compound induced its down-regulation [53].

Two regulatory genes of aflatoxin biosynthesis, *aflR* and *aflS*, which are positive regulator of the whole gene cluster as main activator and enhancer in the regulatory biosynthesis process, respectively, were also inhibited by the exposure to 2-phenylethanol, cinnamaldehyde, citral, eugenol, benzoic acid, and ethanol; they were also inhibited by the essential oils of *Zanthoxylum molle* and *Curcuma longa* and the volatolome of the fungal *Nodulisporium spp*. [64,81,86,88,89,94,95,96]. The volatolome of the bacteria *A. faecalis* and *S. yanglenensis*, as well as the two yeasts *Hanseniaspora sp.*, inhibited the regulation activity of *aflS* and *aflR* [59,67,69].

Some compounds such as benzoic acid or ethanol also showed a reduction in the expression of all the genes involved in the biosynthesis pathway, but this was not explicitly stated [91].

Complete inhibition of aflatoxin production required only 3–4% ethanol. Ethanol at 3.5% and the *A. faecalis* volatolome resulted in down-regulation of all aflatoxin group genes except *aflC* (which controls polyketide synthase) [22,53,59].

As with ethanol, the concentration of cinnamaldehyde always led to a reduction in AFB1 production, but the genes affected were variable. A constant inhibition of *aflT*, which regulates AFB1 secretion, was observed. A specificity was noted at 0.60 mM because the *aflU* was upregulated. In general, with 0.8 mM cinnamaldehyde, 25 of the 30 genes in the aflatoxin group were down-regulated [96]. At a concentration of 0.60 mM, the *aflF* and *aflU* genes were more expressed everywhere, except *aflT, aflS* and *aflR,* compared with the control [96]. Finally, five genes (*aflP, aflC, aflM, aflD, aflT*) were down-regulated by cinnamaldehyde at 0.40 mM [94]. These last five genes were also down-regulated by eugenol at 0.80 mM, whereas only the first three genes were affected by citral 0.56 mM [94,95].

Figure 2 shows that the essential oils of *Zanthoxylum molle* and *Curcuma longa*, as well as *A. faecalis* and *S. yanglenensis*, were the only ones reported to affect the *aflQ* and *aflO* genes involved in the final intermediates of the AFB1 biosynthetic pathway [59,81,86].

When focusing on the modes of action, no successions of mechanisms seem to be attributed to a particular chemical family of VOCs. This could be attributable to the lack of information gathered in this field. However, in general, we can conclude that many VOCs produced by both microorganisms and plants can down-regulate several biosynthetic *afl* genes with different targets and intensity. Therefore, we need more studies to obtain more in-depth knowledge on the links between specific VOCs and specific genes affected.

### 4.3. Impact on the Fungal Growth and Ergosterol Production

The growth of *A. flavus* was proved to be affected by cinnamaldehyde and 2-phenylethanol which completely inhibited the fungal growth [88,96]. Since a related effect on *A. flavus* caused by cinnamaldehyde was the lack of AFB1 production, the fungal physiology and metabolism, particularly the metabolism of certain amino acids required at the hypha apex for fungal growth, were altered [88].

In addition, the volatolomes of species belonging to the *Nodulisporium* genus were shown to interfere with *A. flavus* physiology. In particular, 1,8-cineole inhibited the mitochondrial respiration as well as different stages of mitosis. This last molecule was shown to penetrate through the cell membrane and cause oxidative damage to cell organelles [64]. Finally, among other effects, the essential oil of *Curcuma longa* also induced a considerable reduction in the amount of ergosterol [81].

In summary, although strong effects on the growth of *A. flavus* and its ergosterol production have been shown in some experiments, few studies are available on the functions and mechanisms of VOCs that enable these effects. Therefore, more in-depth investigations are needed to provide the knowledge for possible practical applications of VOCs in the biological control of *A. flavus*. 

## 5. How Can We Exploit These VOCs to our Advantage to Control the Growth of *A. flavus* and Its AFB1 Production?

In the previous paragraphs and in Table 3, Table 4 and Table 5, we have outlined the effects of bioactive VOCs on the growth of *A. flavus* and on the production of AFB1. In order to limit the fungal contamination and AFB1 production, both the early harvesting of maize and quick and controlled storage are recommended [107,108]. However, a further tool that potentially can be integrated in the fight against mycotoxin production at the harvesting phase is the use of bioactive VOCs. Therefore, the selection of bioactive VOCs according to the time of their application in the food chain is also critical to ensuring their antifungal (inhibition of the growth of *A. flavus*) and anti-aflatoxigenic (inhibition of AFB1 production) properties.

Fumigation or pulverization, using bioactive antifungal VOCs could be also considered to dramatically reduce the presence of unfavorable microorganisms on the surface of the grains during harvest and before storage. This approach, which was applied by Sharon et al. (2009) and Hamann et al. (2008), also causes damage to and destruction of the survival structures of the fungus, eventually initiating apoptotic-like cell death [109,110]. However, for a higher efficacy, higher concentrations of VOCs were used by Li et al. (2016) and Tian et al. (2014) [82,86].

In addition, in order to inhibit *A. flavus* growth during storage, using an antifungal compound combined with a selected anti-aflatoxigenic bioactive VOC applied by diffusion could be of interest. In general, fumigation requires a lower concentration than contact, although some exceptions do exist [82,86,90]. Currently, the majority of the bioactive VOCs identified have been shown to have a punctual action due to their fungistatic effect. This means that as soon as the *A. flavus* is no longer subjected to their effects, it regains its virulence and all its faculties to grow and produce AFB1 [61,70]. Therefore, to improve the impact of VOCs on *A. flavus*, setting up a slow diffusion system capable of diffusing the bioactive VOCs over a long period of time would be extremely useful. This objective could be achieved by using new methods of diffusion such as capsules that by a slow release of VOCs in the environment after their dispersion allow a longer temporal dispersion, as proved by Maes et al. (2019) [111]. On the other hand, to apply a bioactive VOC whose effect is permanent would be a reliable alternative. However, it is essential that such a permanent fungicidal effect is effective against all structures of the fungus to avoid any subsequent fungal development after the VOC application.

A further key issue is to optimize the concentration of each VOC since the antifungal efficacy among the bioactive VOCs is highly variable, as shown in vivo experiments over different periods of time on several kinds of food by several authors [77,86,90,93,100]. In addition, such variability has also been confirmed for the VOCs’ anti-aflatoxin activities [81,82,86]. From all these studies, it is evident that, in general, in the vivo experiments a higher concentration was required than in in vitro experiments to completely inhibit *A. flavus* growth and AFB1 production.

Microbial diversity can also be used to inhibit both *A. flavus* growth and AFB1 production, integrating the beneficial action of selected microorganisms that, for example, share the atmosphere of stored grains. All the microorganisms listed in Table 3 have shown a fungistatic effect against *A. flavus*, but only three of them were also investigated for their ability to control and inhibit AFB1 production. The whole volatolomes of *S. saprophyticus* and *A. faecalis* have been tested against other fungal pathogens successfully [59,61]. Dimethyl disulfide, which is one of the major VOCs emitted by these bacteria, is also an effective control, while also promoting plant growth [57,73].

The possible contributions of bioactive VOCs emitted by biological material, such as some crop varieties adapted to local conditions and/or particularly resistant to fungi, have also been shown. Zeringue et al. studied the VOCs emitted by resistant hybrids in order to isolate their specific VOCs and identified mainly aldehydes [92]. Since some maize varieties are less attractive for insects that often are the main vectors of fungal contaminations, the combined use of insect repellent molecules and antifungal complementary bioactive VOCs could be an interesting approach to pursue in future [75].

In addition, since some VOCs have been used as antimicrobial agents in food packaging materials such as polyethylene terephthalate films containing essential oils [78], an extended application of these compounds as new preservation methods could be a further tool to control fungal contamination and mycotoxin production in food packaging.

Finally, it is important to consider that some of the bioactive VOCs discussed here could have negative effects such as possible cytotoxicity for humans and reductions in seed germination, and therefore, these aspects must be well studied before proposing any VOCs use. On the other hand, their volatility leads to an absence of residue on the foodstuff, facilitating its transformation in the food chain since no washing would be required. Thus, unpleasant smells for consumers would be limited, which is an important organoleptic parameter.

In conclusion, the main advantages of using VOCs as bio-control agents are as follows. Firstly, they have a wider and easier diffusion mechanism without requiring physical contact to affect the fungus and there is an absence of residues on the crop. Secondly, an application of bioactive VOCs at key points of the food chain could be an efficient solution to control the fungal growth and, therefore, the production of AFB1 and reduce the use of preservatives that can add unpleasant odors to food. On the other hand, it is necessary to take into account that there is a balance between the fauna and the flora of a given environment and that the eradication of a species such as *A. flavus* can induce a recrudescence of its competitors or other microorganisms. Therefore, the control of the population of *A. flavus*, although worthwhile, should avoid a dramatic increase of other species producing other mycotoxins or causing other diseases in plants.

## 6. Conclusions

VOCs constitute an elementary chain in inter- and intra-species interactions. The great diversity of VOCs emitted by *A. flavus* strains reported in the literature demonstrates that abiotic factors have a great influence on strain VOC profiles. Interesting VOCs have been isolated and identified as bioactive compounds against the growth of *A. flavus* and/or its production of AFB1. However, the mechanisms involved are poorly studied. Nevertheless, some researchers have oriented their investigations towards the aflatoxin gene cluster. In addition, it is evident that a standardization of the environmental parameters that influence the VOCs production is necessary. This would generate a robust knowledge base for our proposed use of VOCs as a reliable biocontrol tool.

## 7. Perspectives

Studies on bioactive VOCs need to consider some issues including the imprecision of certain parameters, such as the application mode, which are often missed in many research studies and have different consequences for the metabolism of *A. flavus*.

A further issue is the accurate evaluation of the effectiveness of bioactive VOCs on the growth or production of AFB1. The control of abiotic parameters, the type and time of exposure, type of contact and strain of *A. flavus* (TS or NTS) targeted are all key aspects to be assessed. Finally, the possibility that specific VOCs could be identified for TS or NTS of *A. flavus* opens significant opportunities for developing reliable markers that can be used for an early identification of strain toxigenicity, which is difficult to achieve using molecular markers due to the variability of NTS in their *afl* gene profiles.

## Figures and Tables

**Table 1:** Overview of literature references concerning the Volatile Organic Compounds (VOCs) emitted by *A. flavus* strains according to the chemical family of these VOCs and the ability of the *A. flavus* strains to produce AFB1 or not.

(a)	Total Number of VOCs	Chemical Family of VOCs Reported in Literature							
		(b) TS VOCs		(c) NTS VOCs		(d) VOCs Shared by TS and NTS		(e) US VOCs	
**Alcohol**	51	De Lucca et al., 2010 De Lucca et al., 2012 Josselin et al., 2021 Müller et al., 2013 Polizzi et al., 2012 Sun et al., 2014 Sun et al., 2016	[34] [35] [36] [37] [38] [39] [40]	De Lucca et al., 2010 Josselin et al., 2021 Jeleń and Wąsowicz, 1998	[34] [36] [41]	De Lucca et al., 2010 Gao et al., 2002 Josselin et al., 2021 Kamiński et al., 1972 Polizzi et al., 2012 Spraker et al., 2014 Sun et al., 2014	[34] [42] [36] [43] [38] [44] [39]	Jeleń and Wąsowicz, 1998 Kamiński et al., 1972	[41] [43]
**Aldehyde**	23	De Lucca et al., 2010 De Lucca et al., 2012 Josselin et al., 2021 Müller et al., 2013 Sun et al., 2014 Sun et al., 2016	[34] [35] [36] [37] [39] [40]	De Lucca et al., 2010 Sun et al., 2014	[34] [39]	De Lucca et al., 2010 Josselin et al., 2021 Sun et al., 2014	[34] [36] [39]		
**Alkane**	96	De Lucca et al., 2010 De Lucca et al., 2012 Josselin et al., 2021 Müller et al., 2013 Spraker et al., 2014	[34] [35] [36] [37] [44]	De Lucca et al., 2010 De Lucca et al., 2012 Josselin et al., 2021 Spraker et al., 2014 Sun et al., 2014	[34] [35] [36] [44] [39]	De Lucca et al., 2010 De Lucca et al., 2012 Farh and Jeon, 2020 Josselin et al., 2021 Sun et al., 2014	[34] [35] [30] [36] [39]	De Lucca et al., 2012	[35]
**Alkene**	65	De Lucca et al., 2010 De Lucca et al., 2012 Jeleń and Wąsowicz, 1998 Josselin et al., 2021 Polizzi et al., 2012 Sun et al., 2016	[34] [35] [41] [36] [38] [40]	De Lucca et al., 2010 De Lucca et al., 2012 Sun et al., 2014	[34] [35] [39]	De Lucca et al., 2012 Josselin et al., 2021	[35] [36]	Jeleń and Wąsowicz, 1998	[41]
**Alkyne**	9	De Lucca et al., 2012	[35]	De Lucca et al., 2012	[35]				
**Amine**	8	De Lucca et al., 2010 De Lucca et al., 2012 Spraker et al., 2014	[34] [35] [44]						
**Amide**	3	De Lucca et al., 2010 De Lucca et al., 2012	[34] [35]						
**Acid**	13	De Lucca et al., 2010 De Lucca et al., 2012 Josselin et al., 2021	[34] [35] [36]	De Lucca et al., 2010 Sun et al., 2014	[34] [39]	Sun et al., 2014	[39]		
**Ester**	20	De Lucca et al., 2010 De Lucca et al., 2012 Josselin et al., 2021	[34] [35] [36]	De Lucca et al., 2010 De Lucca et al., 2012 Sun et al., 2014	[34] [35] [39]	De Lucca et al., 2010	[34]		
**Ether**	1	De Lucca et al., 2012	[35]						
**Furan**	10	De Lucca et al., 2010 De Lucca et al., 2012 Jeleń and Wąsowicz, 1998 Josselin et al., 2021 Sun et al., 2014 Sun et al., 2016	[34] [35] [41] [36] [39] [40]	De Lucca et al., 2012 Sun et al., 2014	[35] [39]	De Lucca et al., 2010 Josselin et al., 2021 Sun et al., 2014	[34] [36] [39]	Jeleń and Wąsowicz, 1998	[41]
**Ketone**	29	De Lucca et al., 2010 De Lucca et al., 2012 Josselin et al., 2021 Spraker et al., 2014 Sun et al., 2016	[34] [35] [36] [44] [40]	De Lucca et al., 2010 De Lucca et al., 2012 Sun et al., 2014	[34] [35] [39]	De Lucca et al., 2010 De Lucca et al., 2012 Gao et al., 2002 Josselin et al., 2021 Kamiński et al., 1972 Sun et al., 2014	[34] [35] [42] [36] [43] [39]	Polizzi et al., 2012	[38]
**Halogen**	4	De Lucca et al., 2010	[34]					Jeleń and Wąsowicz, 1998	[41]
**Terpene**	69	De Lucca et al., 2010 De Lucca et al., 2012 Josselin et al., 2021 Polizzi et al., 2012 Sun et al., 2016 Zeringue et al., 1993	[34] [35] [36] [38] [40] [45]	De Lucca et al., 2012 Sun et al., 2014	[35] [39]	De Lucca et al., 2010 Josselin et al., 2021	[34] [36]	Gao et al., 2002 Pennerman et al., 2016 Polizzi et al., 2012	[42] [46] [38]
**Others**	9	De Lucca et al., 2010 De Lucca et al., 2012 Spraker et al., 2014 Sun et al., 2014	[34] [35] [44] [39]			De Lucca et al., 2010 Josselin et al., 2021	[34] [36]	Gao et al., 2002	[42]

If the chemical family (a) of VOC is specifically emitted by a toxigenic strain (TS) in the article, then the reference will be listed in column (b), if it is specifically emitted by the non-toxigenic strain (NTS), then the reference is listed in column (c), if the VOC is non-specific (NS) to one of the categories, then the reference is listed in column (d), and if the toxigenicity of the strain is unknown (US), then the reference is listed in column (e).

**Table 2:** Summary of the major effects on *A. flavus* growth and AFB1 production of volatolome or bioactive VOCs emitted by microorganisms, essential oils and individual VOC classed by chemical families.

Application Mode		Contact		No Contact	
Source of Bioactive VOCs		Growth of *A. flavus*	AFB1 Production	Growth of *A. flavus*	AFB1 Production
**Microorganisms**	Bacteria	NA	NA	↓	↓/↑
	Yeast	NA	NA	↓	↓
	Fungi	NA	NA	↓	NA/↓
**Essential oil**		↓	↓	↓	NA/↓
**Individual VOC**	Acid	↓	↓	↓	↓
	Alcohol	↓/↑	↓	↓	↓/↑
	Aldehyde	↓	↓	↓	↓
	Alkane	NA	NA	NA	↓
	Ester	↓	↓	↓	↓
	Furan	NA	↓/↑	NA	↓/↑
	Ketone	NA	NA	↓/↑	↓/↑
	Terpene	↓	↓	↓/↑	↓/↑
	Other	NA	NA	NA	↓

(↓) Inhibition; (↑) Augmentation; (NA) no data available.

**Table 5:** Individual VOCs impacting the growth of *A. flavus* and/or its production of AFB1.

(a)	(b) Name	(c) Source		(d) Impact		(e) Application Mode	References	
				Growth	Aflatoxin			
**Alcohol**	**1-Octen-3-ol**	◌	●	-	-	No contact	Singh et al., 2020	[55]
	**2-Buten-1-ol**	◌		NA	-	No contact	Zeringue and McCormick, 1990	[50]
	**2-Butoxy alcohol**	◌		-	NA	No contact	Zeringue and McCormick, 1990	[50]
	**2-Methylbutan-1-ol**	◌	●	-	+	No contact	Braun et al., 2012 Zeringue and McCormick, 1990	[63] [50]
	**2-Phenylethanol**	◌		-	-	No contact Contact	Chang et al., 2015 Gong et al., 2019 Hua et al., 2014	[88] [60] [89]
	**3-Hepten-1-ol**	◌		-	-	No contact	Zeringue and McCormick, 1990	[50]
	**Cis-hex-3-en-1-ol**	◌		-	NA	No contact Contact	Ma et al., 2017	[90]
	**3-Methylbutan-1-ol**	◌	●	-	+	No contact	Braun et al., 2012 Zeringue and McCormick, 1990	[63] [50]
	**Cis-hex-2-en-1-ol**	◌		-	+	No contact Contact	Ma et al., 2017 Zeringue and McCormick, 1990	[90] [50]
	**Decan-1-ol**	◌	●	+	NA	No contact	Zeringue and McCormick, 1990	[50]
	**Ethanol**	◌	●	-	-	Contact	Ren et al., 2020	[53]
	**Heptan-1-ol**	◌		-	-	No contact	Zeringue and McCormick, 1990	[50]
	**Hexan-1-ol**	◌	●	-	-	No contact Contact	Cleveland et al., 2009 Ma et al., 2017	[51] [90]
	**Nonan-1-ol**	◌		+	NA	No contact Contact	Zeringue and McCormick, 1990 Zhang et al., 2021	[50] [54]
	**Octan-3-ol**	◌	●	-	-	No contact	Cleveland et al., 2009	[51]
	**Pentan-1-ol**	◌		+/-	+/-	No contact	Cleveland et al., 2009 Zeringue and McCormick, 1990	[51] [50]
**Acid**	**2-Methylpropanoic acid**	◌	●	-	NA	No contact	Braun et al., 2012	[63]
	**4-Pentanoic acid**	◌		NA	-	No contact	Zeringue and McCormick, 1990	[50]
	**Benzoic acid**	◌	●	-	-	Contact	Moon et al., 2018	[91]
	**Sorbic acid**	◌		-	-	Contact	Moon et al., 2018	[91]
	**Acetic acid**	◌	●	-	-	Contact	Moon et al., 2018	[91]
	**Propionic acid**	◌		-	-	Contact	Moon et al., 2018	[91]
	**Butyric acid**	◌		-	-	Contact	Moon et al., 2018	[91]
**Aldehyde**	**Trans-hept-2-enal**	◌		-	-	No contact	Cleveland et al., 2009 Zeringue and McCormick, 1990 Zeringue et al., 1996	[51] [50] [92]
	**2,4-Hexadienal**	◌		-	-	No contact	Cleveland et al., 2009 Zeringue and McCormick, 1990	[51] [50]
	**(E,E)-2,4-Heptadienal**	◌		-	NA	No contact	Ma and Johnson, 2021	[93]
	**Oct-2-enal**	◌		-	-	No contact	Cleveland et al., 2009 Zeringue and McCormick, 1990 Zeringue et al., 1996	[51] [50] [92]
	**Benzaldehyde**	◌	●	-	-	No contact	Cleveland et al., 2009	[51]
	**Cinnamaldehyde**	◌		-	-	Contact	Liang et al., 2015 Yin et al., 2015 Wang et al., 2019	[94] [95] [96]
	**Citral**	◌		-	-	Contact	Liang et al., 2015	[94]
	**Diethylacetal 2-hexenal**	◌		-	-	No contact	Zeringue and McCormick, 1990	[50]
	**n-Decyl aldehyde**	◌		-	-	No contact	Wright et al., 2000	[97]
	**Furfural**	◌	●	-	NA	No contact	Zeringue, 2000	[98]
	**Heptanal**	◌	●	-	-	No contact	Zeringue and McCormick, 1990 Zeringue et al., 1996	[50] [92]
	**Hexanal**	◌	●	-	-	No contact Contact	Cleveland et al., 2009 Li et al., 2021 Ma et al., 2017 Wright et al., 2000 Zeringue and McCormick, 1990 Zeringue et al., 1996	[51] [99] [90] [97] [50] [92]
	**Nonanal**	◌	●	-	-	No contact	Cleveland et al., 2009	[51]
	**Nonyl aldehyde**	◌		-	-	No contact	Zeringue and McCormick, 1990 Zeringue et al., 1996	[50] [92]
	**Octanal**	◌	●	-	+/-	No contact	Cleveland et al., 2009 Wright et al., 2000 Zeringue and McCormick, 1990 Zeringue et al., 1996	[51] [97] [50] [92]
	**Sorbaldehyde**	◌		-	NA	No contact	Ma and Johnson, 2021	[93]
	**Trans-hex-2-enal**	◌	●	-	-	No contact	Cleveland et al., 2009 De Lucca et al., 2011 Ma et al., 2017 Zeringue and McCormick, 1990 Zeringue et al., 1996	[51] [100] [90] [50] [92]
	**Trans-2-methylbut-2-enal**	◌	●	NA	-	No contact	Moore et al., 2021	[7]
	**Trans-non-2-enal**	◌		-	-	No contact	Zeringue and McCormick, 1990 Zeringue et al., 1996	[50] [92]
**Ester**	**Ethyl acetate**	◌	●	NA	-	No contact	Zeringue and McCormick, 1990	[50]
	**Hexyl acetate**	◌		-	NA	No contact Contact	Ma et al., 2017	[90]
	**Trans-2-hexenyl acetate**	◌		-	NA	No contact Contact	Ma et al., 2017	[90]
**Furan**	**2,3-Dihydrofuran**		●	NA	-	No contact	Moore et al., 2021 Moore et al., 2022	[7] [101]
	**2-Pentylfuran**	◌	●	=	+	No contact	Cleveland et al., 2009	[51]
**Alkane**	**Decane**	◌	●	NA	-	No contact	Moore et al., 2021	[7]
**Ketone**	**Heptan-3-one**	◌		+	+	No contact	Cleveland et al., 2009	[51]
	**Hexan-3-one**	◌		=	-	No contact	Cleveland et al., 2009	[51]
	**Nonan-2-one**	◌	●	-	-	No contact	Zeringue and McCormick, 1990	[50]
	**Octan-3-one**	◌	●	-	-	No contact	Moore et al., 2021 Moore et al., 2022 Zeringue and McCormick, 1990	[7] [101] [50]
	**3-Octen-2-one**	◌	●	-	-	No contact	Cleveland et al., 2009	[51]
	**Pentan-3-one**	◌		NA	+	No contact	Zeringue and McCormick, 1990	[50]
**Terpene**	**Alpha-pinene**	◌	●	+	(-)	No contact	Zeringue and McCormick, 1990	[50]
	**Beta-pinene**	◌		+	(-)	No contact	Zeringue and McCormick, 1990	[50]
	**Camphene**	◌		NA	-	No contact	Zeringue and McCormick, 1990	[50]
	**Carvacrol**	◌		-	NA	Contact	Yin et al., 2015	[95]
	**Eugenol**	◌		-	NA	Contact	Liang et al., 2015	[94]
	**Farnesol**	◌		-	-	Contact	Wang et al., 2014	[102]
	**Limonene**	◌	●	NA	-	No contact	Zeringue and McCormick, 1990	[50]
	**Myrcene**	◌		NA	+	No contact	Zeringue and McCormick, 1990	[50]
	**Ocimene**	◌		NA	+	No contact	Zeringue and McCormick, 1990	[50]
**Other**	**Methyl jasmonate**	◌		NA	-	No contact	Goodrich-Tanrikulu et al., 1995	[103]

(a) Chemical family. (b) IUPAC name. (c) When a VOC is known to be emitted by *A. flavus* species (in accordance with the Table S1) it is indicated by ● symbol. The ◌ symbol is present when the standard VOC was used in the study. (d) Compilation of (-) inhibitory, (+) stimulating, (=) no significant variation or (NA) unavailable data for growth and production of AFB1. (e) Contact type (contact/non-contact).

## Data Availability

Not applicable.

## Supplementary Materials

The following supporting information can be downloaded at https://www.mdpi.com/article/10.3390/ijms232415557/s1.
