# Supplementary material for: Impact of Volatile Organic Compounds on the Growth of Aspergillus flavus and Related Aflatoxin B1 Production: A Review
Source: Int J Mol Sci. 2022 Dec 8;23(24):15557. doi: 10.3390/ijms232415557 (PMC9779742; doi:10.3390/ijms232415557)
Supplement: Supplementary file 1 [file ijms-23-15557-s001.zip › ijms-2023894-supplementary.pdf]

Table S1: Detailed compilation of VOCs known in the literature to be emitted by *A. flavus* sorted by CAS (Chemical Abstracts Service) number.

(a) Name	(b) Cas number	(c) Chemistry family	(d) *Strains*	(e) Reference		(f) Substrate
2,4-dimethylpenta-1,3-diene	1000-87-9	Alkene	T	(De Lucca *et al.*, 2012)	[35]	Non-sterile cracked maize
octa-1,3-diene	1002-33-1	Alkene	T<br>T<br>U	(De Lucca *et al.*, 2010)<br>(Jeleń and Wąsowicz, 1998)<br>(Polizzi *et al.*, 2012)	[34]<br>[41]<br>[38]	Cracked maize<br>Wheat and oats<br>Wallpaper
ethylbenzene	100-41-4	Alkene	U	(Jeleń and Wąsowicz, 1998)	[41]	/
styrene	100-42-5	Alkene	T + NT/T<br>T<br>NT/T<br>U<br>NT<br>T	(De Lucca *et al.*, 2010)<br>(De Lucca *et al.*, 2012)<br>(Josselin *et al.*, 2021)<br>(Jeleń and Wąsowicz, 1998)<br>(Sun *et al.*, 2014)<br>(Sun *et al.*, 2016)	[34]<br>[35]<br>[36]<br>[41]<br>[39]<br>[40]	PDA + sterile cracked maize<br>Cracked maize<br>PDA<br>/<br>Maize media<br>CSA, CDL, MEA, CMA
phenylmethanol	100-51-6	Alcohol	T	(De Lucca *et al.*, 2010)<br>(De Lucca *et al.*, 2012)	[34]<br>[35]	Non-sterile cracked maize<br>Cracked maize
benzaldehyde	100-52-7	Aldehyde	T	(De Lucca *et al.*, 2010)	[34]	Cracked maize
ethyl phenylethanoate	101-97-3	Ester	T<br>T*	(De Lucca *et al.*, 2010)<br>(Josselin *et al.*, 2021)	[34]<br>[36]	Cracked maize<br>PDA
dodecan-2-ol	10203-28-8	Alcohol	T	(De Lucca *et al.*, 2012)	[35]	Non-sterile cracked maize
eremophilene	10219-75-7	Terpene	U<br>T	(Jeleń and Wąsowicz, 1998)<br>(Zeringue, Bhatnagar and Cleveland, 1993)	[41]<br>[45]	/<br>Adye and Mateles liquid medium
2-ethylcyclobutan-1-one	10374-14-8	Ketone	T	(De Lucca *et al.*, 2012)	[35]	Non-sterile cracked maize
benzeacetic acid	103-82-2	Acid	T	(De Lucca *et al.*, 2010)	[34]	Cracked maize
4a,8-dimethyl-2-(prop-1-en-2-yl)-1,2,3,4,4a,5,6,7-octahydronaphthalene	103827-22-1	Terpene	T*	(Josselin *et al.*, 2021)	[36]	PDA
3-phenylpropen-2-al	104-55-2	Aldehyde	NT	(De Lucca *et al.*, 2010)	[34]	PDA
2-ethylhexan-1-ol	104-76-7	Alcohol	NT/T	(De Lucca *et al.*, 2010)	[34]	Cracked maize
ethyl propionate	105-37-3	Ester	T<br>NT/T<br>NT	(De Lucca *et al.*, 2010)<br>(Josselin *et al.*, 2021)<br>(Sun *et al.*, 2014)	[34]<br>[36]<br>[39]	Cracked maize<br>PDA<br>Maize media
ethyl butyrate	105-54-4	Ester	T<br>NT/T	(Josselin *et al.*, 2021)<br>(Sun *et al.*, 2014)	[36]<br>[39]	PDA<br>Maize media
1-(5-methyl-2-furanyl)propan-1-one	10599-69-6	Ketone	T	(De Lucca *et al.*, 2012)	[35]	Non-sterile cracked maize
ethyl heptanoate	106-30-9	Ester	T	(De Lucca *et al.*, 2010)	[34]	Cracked maize
ethyl octanoate	106-32-1	Ester	NT+ T	(De Lucca *et al.*, 2010)	[34]	PDA + Cracked maize
1,4-dimethylbenzene	106-42-3	Alkene	NT	(Sun *et al.*, 2014)	[39]	Maize media
octan-3-one	106-68-3	Ketone	NT<br>NT<br>T<br>NT/T	(De Lucca *et al.*, 2010)]<br>(De Lucca *et al.*, 2012)<br>(Kamiński *et al.*, 1972)<br>(Sun *et al.*, 2014)	[34]<br>[35]<br>[43]<br>[38]	PDA<br>Non-sterile cracked maize<br>Wheat meal sterilized<br>Maize media
2-methylpentane	107-83-5	Alkane	T<br>T	(De Lucca *et al.*, 2012)<br>(Sun *et al.*, 2014)	[35]<br>[39]	Non-sterile cracked maize<br>Maize media
pentan-2-one	107-87-9	Ketone	NT/T	(Sun *et al.*, 2014)	[39]	Maize media
1,3-dimethylbenzene	108-38-3	Alkene	T	(De Lucca *et al.*, 2012)	[35]	Non-sterile cracked maize
ethyl 3-methylbutyrate	108-64-5	Ester	NT/T	(Sun *et al.*, 2014)	[39]	Maize media
1,3,5-trimethylbenzene	108-67-8	Alkene	T	(De Lucca *et al.*, 2012)	[35]	Non-sterile cracked maize

methylcyclohexane	108-87-2	Alkane	T	(De Lucca *et al*., 2012)	[35]	Sterile cracked maize
toluene	108-88-3	Alkene	T<br>T<br>NT<br>T	(De Lucca *et al*., 2010)<br>(Josselin *et al*., 2021)<br>(Sun *et al*., 2014)<br>(Sun *et al*., 2016)	[34]<br>[36]<br>[39]<br>[40]	Craked maize<br>PDA<br>Maize media<br>CDA, CSA, CDL, MEA,
aromadrendrene	109119-91-7	Terpene	U<br>T	(Jeleń and Wąsowicz, 1998)<br>(Josselin *et al*., 2021)	[41]<br>[36]	/<br>PDA
pentane	109-66-0	Alkane	NT/T	(Sun *et al*., 2014)	[39]	Maize media
tetrahydrofuran	109-99-9	Furan	T<br>T	(De Lucca *et al*., 2010)<br>(De Lucca *et al*., 2012)	[34]<br>[35]	Cracked maize<br>Non-sterile cracked maize
furan	110-00-9	Furan	NT/T	(Sun *et al*., 2014)	[39]	Maize media
propan-2-yl tetradecanoate	110-27-0	Ester	T	(De Lucca *et al*., 2010)	[34]	Cracked maize
alpha-cedrene	11028-42-5	Terpene	U	(Polizzi *et al*., 2012)	[38]	Wallpaper
heptan-2-one	110-43-0	Ketone	U<br>U<br>NT/T	(Gao *et al*., 2002) *<br>(Polizzi *et al*., 2012)<br>(Sun *et al*., 2014)	[39]<br>[38]<br>[39]	Gypsum board<br>Malt extract agar<br>Maize media
hexane	110-54-3	Alkane	T<br>T<br>NT/T	(De Lucca *et al*., 2010)<br>(De Lucca *et al*., 2012)<br>(Josselin *et al*., 2021)	[34]<br>[35]<br>[36]	PDA<br>Non-sterile cracked maize<br>PDA
pentanal	110-62-3	Aldehyde	NT	(Sun *et al*., 2014)	[39]	Maize media
octan-2-one	111-13-7	Ketone	NT/T	(Sun *et al*., 2014)	[39]	Maize media
hexan-1-ol	111-27-3	Alcohol	NT/T<br>T	(Spraker *et al*., 2014)<br>(De Lucca *et al*., 2012)	[44]<br>[35]	Maize media<br>Non-sterile cracked maize
butoxyethene	111-34-2	Ether	T	(De Lucca *et al*., 2012)	[35]	Non-sterile cracked maize
2-methylbut-2-enal	1115-11-3	Aldehyde	NT<br>NT<br>NT/T	(De Lucca *et al*., 2010)<br>(De Lucca *et al*., 2012)<br>(Josselin *et al*., 2021)	[34]<br>[35]<br>[36]	PDA<br>Non-sterile cracked maize<br>PDA
octane	111-65-9	Alkane	NT/T<br>T<br>NT/T	(De Lucca *et al*., 2010)<br>(Josselin *et al*., 2021)<br>(Sun *et al*., 2014)	[34]<br>[36]<br>[39]	Cracked maize<br>PDA<br>Maize media
oct-1-ene	111-66-0	Alkene	T<br>T	(De Lucca *et al*., 2010)<br>(De Lucca *et al*., 2012)	[34]<br>[35]	Cracked maize<br>Non-sterile cracked maize
oct-2-ene	111-67-1	Alkene	T	(De Lucca *et al*., 2012)	[35]	Non-sterile cracked maize
2-butoxyethanol	111-76-2	Alcohol	T	(De Lucca *et al*., 2010)	[34]	Cracked maize
nonane	111-84-2	Alkane	NT/T	(De Lucca *et al*., 2010)	[34]	Cracked maize
octan-1-ol	111-87-5	Alcohol	U	(Jeleń and Wąsowicz, 1998)	[41]	/
2-(2-ethoxyethoxy)-ethanol	111-90-0	Alcohol	T	(De Lucca *et al*., 2010)	[34]	Cracked maize
dodec-1-ene	1120-36-1	Alkene	T*	(Josselin *et al*., 2021)	[36]	PDA
nonanoic acid	112-05-0	Acid	T	(De Lucca *et al*., 2010)	[34]	Cracked maize
heptyl acetate	112-06-1	Ester	T	(De Lucca *et al*., 2012)	[35]	Non-sterile cracked maize
2-methylcyclopent-2-en-1-one	1120-73-6	Ketone	T	(De Lucca *et al*., 2012)	[35]	Sterile cracked maize
2-methylcyclohex-2-en-1-one	1121-18-2	Ketone	T	(De Lucca *et al*., 2012)	[35]	Non-sterile cracked maize
decan-1-ol	112-30-1	Alcohol	T	(Josselin *et al*., 2021)	[36]	PDA
decanal	112-31-2	Aldehyde	T<br>T	(De Lucca *et al*., 2010)<br>(Sun *et al*., 2016)	[34]<br>[40]	Cracked maize<br>CDA, CSA, CDL, MEA, CMA
dodecane	112-40-3	Alkane	NT/T<br>T	(De Lucca *et al*., 2010)<br>(Sun *et al*., 2016)	[34]<br>[40]	Cracked maize<br>CDA, CSA, CDL, MEA, CMA

13-docosenamide	112-84-5	Amide	T	(De Lucca *et al.*, 2010)	[34]	PDA
eicosane	112-95-8	Alkane	NT/T	(De Lucca *et al.*, 2010)	[34]	Cracked maize
propene	115-07-1	Alkene	T	(De Lucca *et al.*, 2012)	[35]	Sterile cracked maize
beta-humulene	116-04-1	Terpene	T	(Sun *et al.*, 2016)	[40]	CSA, CDL, MEA
2-methylbutanoic acid	116-53-0	Acid	NT	(Sun *et al.*, 2014)	[39]	Maize media
(Z)-1,2-dimethylcyclopentane	1192-18-3	Alkane	T	(De Lucca *et al.*, 2012)	[35]	Sterile cracked maize
benzeneacetaldehyde	122-78-1	Aldehyde	T	(De Lucca *et al.*, 2010)	[34]	Cracked maize
2-phenoxyethanol	122-99-6	Alcohol	T<br>U	(De Lucca *et al.*, 2010)<br>(Polizzi *et al.*, 2012)	[34]<br>[38]	Cracked maize<br>Wallpaper
4-ethylphenol	123-07-9	Alcohol	T	(De Lucca *et al.*, 2012)	[35]	Non-sterile cracked maize
3-methylbutan-1-ol	123-51-3	Alcohol	NT/T<br>U<br>U<br>NT/T<br>NT/T<br>T	(De Lucca *et al.*, 2010)<br>(Gao *et al.*, 2002)<br>(Jeleń and Wąsowicz, 1998)<br>(Josselin *et al.*, 2021)<br>(Sun *et al.*, 2014)<br>(Zeringue, Bhatnagar and Cleveland, 1993)	[34]<br>[42]<br>[41]<br>[36]<br>[39]<br>[45]	PDA + cracked maize<br>Gypsum board<br>/<br>Maize media<br>PDA<br>MEA + Gypsum board
ethyl hexanoate	123-66-0	Ester	T	(De Lucca *et al.*, 2010)	[34]	Cracked maize
non-1-ene	124-11-8	Alkene	T<br>T	(De Lucca *et al.*, 2010)<br>(De Lucca *et al.*, 2012)	[34]<br>[35]	Cracked maize<br>Non-sterile cracked maize
octanal	124-13-0	Aldehyde	T<br>T	(De Lucca *et al.*, 2010)<br>(De Lucca *et al.*, 2012)	[34]<br>[35]	Cracked maize<br>Non-sterile cracked maize
decane	124-18-5	Alkane	NT<br>T<br>NT	(De Lucca *et al.*, 2010)<br>(De Lucca *et al.*, 2012)<br>(Sun *et al.*, 2014)	[34]<br>[35]<br>[39]	Cracked maize<br>Non-sterile cracked maize<br>Maize media
nonanal	124-19-6	Aldehyde	NT/T	(De Lucca *et al.*, 2010)	[34]	Cracked maize
isobornyl acetate	125-12-2	Ester	T	(De Lucca *et al.*, 2010)	[34]	Cracked maize
2,6-dimethyldecane	13150-81-7	Alkane	T	(De Lucca *et al.*, 2012)	[35]	Sterile cracked maize
4-methyldec-1-ene	13151-29-6	Alkene	T	(De Lucca *et al.*, 2012)	[35]	Non-sterile cracked maize
3a,4,7,7,7a-Tetrahydro-4,7-methano-1H-indene	13257-74-4	Terpene	T	(De Lucca *et al.*, 2010)	[34]	Cracked maize
6-methyltridecane	13287-21-3	Alkane	T	(De Lucca *et al.*, 2012)	[35]	Non-sterile cracked maize
pentadec-1-ene	13360-61-7	Alkene	T	(De Lucca *et al.*, 2012)	[35]	Non-sterile cracked maize
(E)-alpha-bergamotene	13474-59-4	Terpene	T	(Sun *et al.*, 2016)	[40]	CDA, CSA, CDL, MEA, CMA
9-octyleicosane	13475-77-9	Alkane	NT	(De Lucca *et al.*, 2012)	[35]	Non-sterile cracked maize
2,2,4,6,6-pentamethylheptane	13475-82-6	Alkane	NT/T	(Josselin *et al.*, 2021)	[36]	PDA
2,5-dimethylcyclohexa-2,5-diene-1,4-dione	137-18-8	Ketone	T	(De Lucca *et al.*, 2012)	[35]	Sterile cracked maize
2-methylbutan-1-ol	137-32-6	Alcohol	NT/T<br>U<br>NT/T<br>U	(De Lucca *et al.*, 2010)<br>(Gao *et al.*, 2002)<br>(Josselin *et al.*, 2021)<br>(Spraker *et al.*, 2014)	[34]<br>[39]<br>[36]<br>[44]	Cracked maize<br>MEA<br>PDA<br>Maize media
beta-cubebene	13744-15-5	Terpene	U<br>T<br>T	(Jeleń and Wąsowicz, 1998)<br>(Sun *et al.*, 2016)<br>(Zeringue, Bhatnagar and Cleveland, 1993)	[41]<br>[40]<br>[45]	/<br>CDA, CSA, CDL, CMA<br>Adye and Mateles liquid medium
limonene	138-86-3	Terpene	T<br>T<br>U<br>T	(De Lucca *et al.*, 2010)<br>(De Lucca *et al.*, 2012)<br>(Jeleń and Wąsowicz, 1998)<br>(Sun *et al.*, 2016)	[34]<br>[35]<br>[41]<br>[40]	Cracked maize<br>Sterile cracked maize<br>/<br>CSA, CDL, MEA, CMA

ethyl acetate	141-78-6	Ester	NT+T<br>NT<br>NT/T<br>NT/T	(De Lucca *et al.*, 2010)<br>(De Lucca *et al.*, 2012)<br>(Sun *et al.*, 2014)<br>(Josselin *et al.*, 2021)	[34]<br>[35]<br>[39]<br>[36]	PDA + cracked maize<br>Sterile cracked maize<br>Maize media<br>PDA
1,3-diethylbenzene	141-93-5	Alkene	T	(De Lucca *et al.*, 2012)	[35]	Non-sterile cracked maize
ethyl acetoacetate	141-97-9	Ester	T	(De Lucca *et al.*, 2012)	[35]	Sterile cracked maize
heptane	142-82-5	Alkane	NT/T<br>NT/T<br>NT/T	(De Lucca *et al.*, 2010)<br>(Josselin *et al.*, 2021)<br>(Sun *et al.*, 2014)	[34]<br>[36]<br>[39]	Cracked maize<br>PDA<br>Maize media
(2E,4E)-hexa-2,4-dienal	142-83-6	Aldehyde	T<br>T	(De Lucca *et al.*, 2010)<br>(De Lucca *et al.*, 2012)	[34]<br>[35]	Non-sterile cracked maize<br>Sterile cracked maize
(Z)-9-octadecen-1-ol	143-28-2 cis	Alcohol	T	(De Lucca *et al.*, 2010)	[34]	Cracked maize
2,3,3-trimethylcyclobutanone	1449-49-6	Ketone	T	(De Lucca *et al.*, 2012)	[35]	Non-sterile cracked maize
cadina-1(10),6,8-triene	1460-96-4	Terpene	T	(Sun *et al.*, 2016)	[40]	CDA, CSA, CDL, MEA
beta-himachalene	1461-03-6	Terpene	NT/T<br>U	(Josselin *et al.*, 2021)<br>(Polizzi *et al.*, 2012)	[36]<br>[38]	PDA<br>Wallpaper
2,6,10-trimethyltetradecane	14905-56-7	Alkane	T	(De Lucca *et al.*, 2010)	[34]	Cracked maize
ylangene	14912-44-8	Terpene	T	(Sun *et al.*, 2016)	[40]	CDA, CSA, CDL, MEA
methylcyclooctane	1502-38-1	Alkane	T<br>T*	(De Lucca *et al.*, 2012)<br>(Josselin *et al.*, 2021)	[35]<br>[36]	Non-sterile cracked maize<br>PDA
bicyclo[4.4.0]dec-1-en,2-isopropyl-5-methyl-9-methylene	150320-52-8	Terpene	T<br>T	(Sun *et al.*, 2016)<br>(Zeringue, Bhatnagar and Cleveland, 1993)	[40]<br>[45]	CDA, CDL, MEA, CMA<br>Adye and Mateles liquid medium
5,5-dimethylhexa-1,3-diene	1515-79-3	Alkene	NT	(De Lucca *et al.*, 2010)	[34]	PDA
2-methyloctadecane	1560-88-9	Alkane	T	(De Lucca *et al.*, 2012)	[35]	Non-sterile cracked maize
2-methylheptadecane	1560-89-0	Alkane	T	(De Lucca *et al.*, 2012)	[35]	Non-sterile cracked maize
(Z)-muurola-3,5-diene	157374-44-2	Terpene	T	(Josselin *et al.*, 2021)	[36]	PDA
4-ethyloctane	15869-86-0	Alkane	T	(De Lucca *et al.*, 2012)	[35]	Non-sterile cracked maize
2,5-dimethyloctane	15869-89-3	Alkane	T	(De Lucca *et al.*, 2012)	[35]	Non-sterile cracked maize
docos-1-ene	1599-67-3	Alkene	T	(De Lucca *et al.*, 2012)	[35]	Non-sterile cracked maize
2,3-dimethylpenta-1,3-diene	1625-49-6	Alkene	NT	(De Lucca *et al.*, 2012)	[35]	Non-sterile cracked maize
1,1-dimethylcyclopentane	1630-94-0	Alkane	T	(De Lucca *et al.*, 2012)	[35]	Non-sterile cracked maize
5-methylundecane	1632-70-8	Alkane	T	(De Lucca *et al.*, 2012)	[35]	Non-sterile cracked maize
4-ethyldecane	1636-44-8	Alkane	T	(De Lucca *et al.*, 2012)	[35]	Non-sterile cracked maize
undecan-2-ol*	1653-30-1	Alcohol	T	(De Lucca *et al.*, 2012)	[35]	Non-sterile cracked maize
1-methyl-3-prop-1-en-2-ylcyclohexene	16580-24-8	Alkene	T	(De Lucca *et al.*, 2012)	[35]	Non-sterile cracked maize
ethylcyclohexane	1678-91-7	Alkane	T	(De Lucca *et al.*, 2012)	[35]	Non-sterile cracked maize
heptadecan-2-ol	16813-18-6	Alcohol	T	(De Lucca *et al.*, 2012)	[35]	Non-sterile cracked maize
alpha-cuparene	16982-00-6	Terpene	U	(Polizzi *et al.*, 2012)	[38]	Wallpaper
beta-selinene	17066-67-0	Terpene	T	(Josselin *et al.*, 2021)	[36]	PDA
2,6-dimethylundecane	17301-23-4	Alkane	T	(De Lucca *et al.*, 2012)	[35]	Non-sterile cracked maize
4,5-dimethylnonane	17302-23-7	Alkane	T<br>T	(De Lucca *et al.*, 2010)<br>(De Lucca *et al.*, 2012)	[34]<br>[35]	Cracked maize<br>Non-sterile cracked maize
2,5-dimethylnonane	17302-27-1	Alkane	T	(De Lucca *et al.*, 2010)	[34]	Cracked maize
2,6-dimethylnonane	17302-28-2	Alkane	T	(De Lucca *et al.*, 2012)	[35]	Non-sterile cracked maize
2,5-dimethyldecane	17312-50-4	Alkane	T	(De Lucca *et al.*, 2010)	[34]	Cracked maize
3,7-dimethyldecane	17312-54-8	Alkane	T	(De Lucca *et al.*, 2012)	[35]	Non-sterile cracked maize
alpha.-cubebene	17699-14-8	Terpene	T	(De Lucca *et al.*, 2012)	[35]	Non-sterile cracked maize

			T<br>T	(Josselin *et al.*, 2021)<br>(Sun *et al.*, 2016)	[36]<br>[40]	PDA<br>CDA, CSA, CDL, MEA, CMA
p-mentha-1,3,8-triene	18368-95-1	Terpene	NT	(De Lucca *et al.*, 2012)	[35]	Non-sterile cracked maize
beta-chamigrene	18431-82-8	Terpene	T*	(Josselin *et al.*, 2021)	[36]	PDA
5-methylhexanal	1860-39-5	Aldehyde	T	(De Lucca *et al.*, 2012)	[35]	Non-sterile cracked maize
hept-2-enal	18829-55-5	Aldehyde	T<br>NT	(De Lucca *et al.*, 2012)<br>(Sun *et al.*, 2014)	[35]<br>[39]	Non-sterile cracked maize<br>Maize media
hexacos-1-ene	18835-33-1	Alkene	T	(De Lucca *et al.*, 2012)	[35]	Non-sterile cracked maize
dec-3-ene	19398-37-9	Alkene	T	(De Lucca *et al.*, 2012)	[35]	Non-sterile cracked maize
3-dodec-2-enyloxolane-2,5-dione	19780-11-1	Ketone	T	(De Lucca *et al.*, 2010)	[34]	Cracked maize
tau-muurolol	19912-62-0	Terpene	T*	(Josselin *et al.*, 2021)	[36]	PDA
epi-cubeno-1-ol	19912-67-5	Terpene	T	(Josselin *et al.*, 2021)	[36]	PDA
alpha-chamigrene	19912-83-5	Terpene	U	(Polizzi *et al.*, 2012)	[38]	Wallpaper
alpha-corocalene	20129-39-9	Terpene	T*	(Josselin *et al.*, 2021)	[36]	PDA
1-2-(2-methyloxy-1-methylethoxy)-1-methylethoxy]-propan-2-ol	20324-33-8	Alcohol	T	(De Lucca *et al.*, 2010)	[34]	Cracked maize
1-iodododecane	2050-77-3	Halogen	T	(De Lucca *et al.*, 2010)	[34]	Cracked maize
2-ethylbut-2-enal	20521-42-0	Aldehyde	T	(De Lucca *et al.*, 2012)	[35]	Non-sterile cracked maize
2,2-dimethylheptane-3,5-dione	20734-29-6	Ketone	T	(Sun *et al.*, 2016)	[40]	CSA, CDL, MEA, CMA
4-methylpent-2-yne	21020-27-9	Alkyne	T	(De Lucca *et al.*, 2012)	[35]	Non-sterile cracked maize
2-octadecoxyethanol	2136-72-3	Alcohol	T	(De Lucca *et al.*, 2012)	[35]	Non-sterile cracked maize
alpha-calacorene	21391-99-1	Terpene	T	(Josselin *et al.*, 2021)	[36]	PDA
4-methyloctane	2216-34-4	Alkane	T<br>T	(De Lucca *et al.*, 2010)<br>(De Lucca *et al.*, 2012)	[34]<br>[35]	Cracked maize<br>Non-sterile cracked maize
gamma-gurjunene	22567-17-5	Terpene	T<br>U<br>U	(Josselin *et al.*, 2021)<br>(Jeleń and Wąsowicz, 1998)<br>(Zeringue, Bhatnagar and Cleveland, 1993)	[36]<br>[41]<br>[45]	PDA<br>/<br>Adye and Mateles liquid medium
1-hepten-3-yne	2384-73-8	Alkyne	NT	(De Lucca *et al.*, 2012)	[35]	Non-sterile cracked maize
germacrene-d	23986-74-5	Terpene	T<br>T	(Josselin *et al.*, 2021)<br>(Sun *et al.*, 2016)	[36]<br>[40]	PDA<br>CDA, CSA, CDL, MEa
n-methyloctan-1-amine	2439-54-5	Amine	T	(De Lucca *et al.*, 2012)	[35]	Non-sterile cracked maize
alpha-cadinene	24406-05-1	Terpene	T	(Josselin *et al.*, 2021)	[36]	PDA
1,2-dimethylcyclopene	2452-99-5	Alkene	T	(De Lucca *et al.*, 2012)	[35]	Non-sterile cracked maize
(3E)-3-methylhexa-1,3,5-triene	24587-26-6	Alkene	T	(De Lucca *et al.*, 2012)	[35]	Non-sterile cracked maize
2-methylhexadecan-1-ol	2490-48-4	Alcohol	T	(De Lucca *et al.*, 2012)	[35]	Non-sterile cracked maize
5-methyltridecane	25117-31-1	Alkane	T	(De Lucca *et al.*, 2012)	[35]	Non-sterile cracked maize
(Z)-1,3-dimethylcyclopentane	2532-58-3	Alkane	T	(De Lucca *et al.*, 2012)	[35]	Sterile cracked maize
(E)-2-octanal	2548-87-0	Aldehyde	T	(Müller *et al.*, 2013)	[37]	Maize silk
hept-3-yne	2586-89-2	Alkyne	T	(De Lucca *et al.*, 2012)	[35]	Sterile cracked maize
(Z)-oct-2-en-1-ol	26001-58-1	Alcohol	T<br>U	(De Lucca *et al.*, 2010)<br>(Jeleń and Wąsowicz, 1998)	[34]<br>[41]	Cracked maize<br>/
tetrahydro-6,6-dimethyl-2h-pyran-2-one	2610-95-9	Ketone	T	(De Lucca *et al.*, 2012)	[35]	Non-sterile cracked maize
4-methylhept-6-en-3-one	26118-97-8	Ketone	T	(Spraker *et al.*, 2014)	[44]	Glucose minimal medium (GMM)
4-methylheptadecane	26429-11-8	Alkane	T	(De Lucca *et al.*, 2012)	[35]	Non-sterile cracked maize
7-methyltridecane	26730-14-3	Alkane	T	(De Lucca *et al.*, 2012)	[35]	Non-sterile cracked maize
(Z)-hexadec-2-ene	26741-29-7	Alkene	T	(Sun *et al.*, 2016)	[40]	CDA, CSA, MEA, CMA
4,4-dimethyl-1,2-pentadiene	26981-77-1	Alkene	T	(De Lucca *et al.*, 2012)	[35]	Non-sterile cracked maize

1,2-benzisothiazole	272-16-2	Other	T	(De Lucca *et al.*, 2010)	[34]	Cracked maize
cyclopropene	2781-85-3	Alkene	T<br>T	(De Lucca *et al.*, 2012)<br>(De Lucca *et al.*, 2010)	[35]<br>[34]	Sterile cracked maize<br>Non-sterile cracked maize
1-methoxybut-1-en-3-yne	2798-73-4	Alkyne	T	(De Lucca *et al.*, 2012)	[35]	Non-sterile cracked maize
hexa-2,4-diyne	2809-69-0	Alkyne	T	(De Lucca *et al.*, 2012)	[35]	Non-sterile cracked maize
4-methyldecane	2847-72-5	Alkane	NT/T	(De Lucca *et al.*, 2010)	[34]	Cracked maize
beta-acoradiene	28477-64-7	Terpene	U	(Polizzi *et al.*, 2012)	[38]	Wallpaper
gamma-selinene	28624-23-9	Terpene	U<br>T	(Jeleń and Wąsowicz, 1998)<br>(Zeringue, Bhatnagar and Cleveland, 1993)	[41]<br>[45]	/<br>Adye and Mateles liquid medium
1,3-dimethyl-2-ethylbenzene	2870-04-4	Alkene	U	(Jeleń and Wąsowicz, 1998)	[41]	/
3-methylhexa-2,4-diene	28823-42-9	Alkene	T	(De Lucca *et al.*, 2012)	[35]	Non-sterile cracked maize
gamma-curcumene	28976-68-3	Terpene	U	(Polizzi *et al.*, 2012)	[38]	Wallpaper
cyclobutanol	2919-23-5	Alcohol	T	(De Lucca *et al.*, 2012)	[35]	Sterile cracked maize
cyclodecane	293-96-9	Alkane	T	(De Lucca *et al.*, 2010)	[34]	Cracked maize
cyclododecane	294-62-2	Alkane	T	(De Lucca *et al.*, 2010)	[34]	Cracked maize
4-methylundecane	2980-69-0	Alkane	T	(De Lucca *et al.*, 2010)	[34]	Cracked maize
o-decylhydroxylamine	29812-79-1	Amine	T	(De Lucca *et al.*, 2012)	[35]	Non-sterile cracked maize
gamma-muurolene	30021-74-0	Terpene	U<br>T	(Jeleń and Wąsowicz, 1998)<br>(Josselin *et al.*, 2021)	[41]<br>[36]	/<br>PDA
(Z)-9-octadecenamide	301-02-0	Amide	T	(De Lucca *et al.*, 2010)	[34]	PDA
2,6,11-trimethyldodecane	31295-56-4	Alkane	T<br>T	(De Lucca *et al.*, 2010)<br>(De Lucca *et al.*, 2012)	[34]<br>[35]	Cracked maize<br>Non-sterile cracked maize
alpha-muurolene	31983-22-9	Terpene	T<br>T	(Zeringue, Bhatnagar and Cleveland, 1993)<br>(Josselin *et al.*, 2021)	[45]<br>[36]	Adye and Mateles liquid medium<br>PDA
2-ethylfuran	3208-16-0	Furan	T<br>T	(De Lucca *et al.*, 2012)<br>(Sun *et al.*, 2014)	[35]<br>[39]	Non-sterile cracked maize<br>Maize media
2,4,5-trimethyl-1,3-dioxolane	3299-32-9	Other	NT/T	(Josselin *et al.*, 2021)	[36]	PDA
decanoic acid	334-48-5	Acid	T	(De Lucca *et al.*, 2010)	[34]	Cracked maize
1-octen-3-ol	3391-86-4	Alcohol	T<br>U<br>NT/T	(Kamiński *et al.*, 1972),<br>(Polizzi *et al.*, 2012)*<br>(Sun *et al.*, 2014)	[43]<br>[38]<br>[39]	/<br>Malt extract agar<br>Maize media
(Z)-hexadec-3-ene	34303-81-6	Alkene	T	(Sun *et al.*, 2016)	[40]	CDA, CSA, CDL, MEA, CMA
thiochroman-4-one	3528-17-4	Ketone	T*	(Josselin *et al.*, 2021)	[36]	PDA
(E)-hexadec-7-ene	35507-09-6	Alkene	T	(Sun *et al.*, 2016)	[40]	CSA, CDL, MEA, CMA
2-methoxyethylbenzene	3558-60-9	Alkene	T	(De Lucca *et al.*, 2012)	[35]	Non-sterile cracked maize
alpha-guaiene	3691-12-1	Terpene	T	(Sun *et al.*, 2016)	[40]	CDA, CSA, CDL, CMA
2,4-dimethylfuran	3710-43-8	Furan	T	(Sun *et al.*, 2014)	[39]	Maize media
2-nonenoic acid*	3760-11-0	Acid	T	(De Lucca *et al.*, 2012)	[35]	Non-sterile cracked maize
2-pentylfuran	3777-69-3	Furan	T<br>NT/T	(De Lucca *et al.*, 2010)<br>(Sun *et al.*, 2014)	[34]<br>[39]	Cracked maize<br>Maize media
5,6,7-trimethoxy-2,3-dihydroinden-1-one	38472-90-1	Ketone	T	(De Lucca *et al.*, 2010)	[34]	Cracked maize
alpha-copaene	3856-25-5	Terpene	U<br>T<br>T	(Jeleń and Wąsowicz, 1998)<br>(Josselin *et al.*, 2021)<br>(Zeringue, Bhatnagar and Cleveland, 1993)	[41]<br>[36]<br>[45]	/<br>PDA<br>Adye and Mateles liquid medium
gamma-cadinene	39029-41-9	Terpene	T<br>T<br>T	(Josselin *et al.*, 2021)<br>(Polizzi *et al.*, 2012)*<br>(Zeringue, Bhatnagar and Cleveland, 1993)	[36]<br>[38]<br>[45]	PDA<br>Adye and Mateles liquid medium,<br>Malt extract agar

2-butyloctan-1-ol	3913-02-8	Alcohol	T	(Josselin *et al.*, 2021)	[36]	PDA
(2E,5E)-hepta-2,5-diene	39619-60-8	Alkene	T	(De Lucca *et al.*, 2012)	[35]	Non-sterile cracked maize
1,3-cycloheptadiene	4054-38-0	Alkene	T	(De Lucca *et al.*, 2012)	[35]	Non-sterile cracked maize
4-methoxy-2,5-dimethylfuran-3-one	4077-47-8	Ketone	T	(De Lucca *et al.*, 2010)	[34]	Cracked maize
epizonaren	41702-63-0	Terpene	NT<br>U<br>T<br>T	(De Lucca *et al.*, 2012)<br>(Jeleń and Wąsowicz, 1998)<br>(Josselin *et al.*, 2021)<br>(Zeringue, Bhatnagar and Cleveland, 1993)	[35]<br>[41]<br>[36]<br>[45]	Non-sterile cracked maize<br>/<br>PDA<br>Adye and Mateles liquid medium
but-2-enal	4170-30-3	Aldehyde	T	(De Lucca *et al.*, 2012)	[35]	Sterile cracked maize
eicos-9-ene	42448-90-8	Alkene	T	(De Lucca *et al.*, 2012)	[35]	Non-sterile cracked maize
1,1,2-trimethylcyclopentane	4259-00-1	Alkane	T	(De Lucca *et al.*, 2012)	[35]	Non-sterile cracked maize
butan-2,3-dione	431-03-8	Ketone	NT/T	(Sun *et al.*, 2014)	[39]	Maize media
2-heptyl-1,3-dioxolane	4359-57-3	Other	T	(Spraker *et al.*, 2014)	[44]	Glucose minimal medium (GMM)
mono(2-ethylhexyl) phthalate	4376-20-9	Acid	T	(De Lucca *et al.*, 2010)	[34]	Cracked maize
2-butylfuran	4466-24-4	Furan	T	(De Lucca *et al.*, 2012)	[35]	Sterile cracked maize
valencene	4630-07-3	Terpene	T<br>T*<br>T<br>T	(Jeleń and Wąsowicz, 1998)<br>(Josselin *et al.*, 2021)<br>(Sun *et al.*, 2016)<br>(Zeringue, Bhatnagar and Cleveland, 1993)	[41]<br>[36]<br>[40]<br>[45]	/<br>PDA<br>CDA, CSA, CDL, MEA, CMA<br>Adye and Mateles liquid medium
3,3-dimethylbutane-2-ol	464-07-3	Alcohol	T	(De Lucca *et al.*, 2012)	[35]	Non-sterile cracked maize
cedrene	469-61-4	Terpene	T	(De Lucca *et al.*, 2012)	[35]	Non-sterile cracked maize
thujopsene	470-40-6 (cis)	Terpene	U	(Jeleń and Wąsowicz, 1998)	[41]	/
1-methyl-4-propan-2-yl-7-oxabicyclo2.2.1]heptane (1,4-Cineole)	470-67-7	Alkane	T	(De Lucca *et al.*, 2010)	[34]	Cracked maize
eucalyptol	470-82-6	Terpene	T	(De Lucca *et al.*, 2010)	[34]	Cracked maize
alpha-selinene	473-13-2	Terpene	T<br>T	(Josselin *et al.*, 2021)<br>(Sun *et al.*, 2016)	[36]<br>[40]	PDA<br>CDA, CSA, CDL, MEA
alpha-cadinol	481-34-5	Terpene	T<br>T	(Josselin *et al.*, 2021)<br>(Sun *et al.*, 2016)	[36]<br>[40]	PDA<br>CDA, CSA, CDL, MEA
delta-cadinene	483-76-1	Terpene	U<br>NT/T<br>U<br>T<br>T	(Jeleń and Wąsowicz, 1998)<br>(Josselin *et al.*, 2021)<br>(Polizzi *et al.*, 2012)<br>(Sun *et al.*, 2016)<br>(Zeringue, Bhatnagar and Cleveland, 1993)	[41]<br>[36]<br>[38]<br>[40]<br>[45]	/<br>PDA<br>MEA<br>CSA, CDL, MEA, CMA<br>Adye and Mateles liquid medium
1,6-dimethyl-4-(1-methylethyl)naphthalene	483-78-3	Alkene	T	(Sun *et al.*, 2016)	[40]	CSA, CDL, CMA
1-cyclohexene-1-methanol	4845-04-9	Alkene	T	(De Lucca *et al.*, 2012)	[35]	Non-sterile cracked maize
tetramethylbenzene	488-23-3	Alkene	T<br>T	(Jeleń and Wąsowicz, 1998)<br>(De Lucca *et al.*, 2012)	[41]	Agar<br>Non-sterile cracked maize
6,10,11,11-tetramethyltricyclo6.3.0.1e2,3]undec-1(7)ene	489-39-4	Terpene	T	(Zeringue, Bhatnagar and Cleveland, 1993)	[45]	Adye and Mateles liquid medium
alpha-gurjunene	489-40-7	Terpene	U<br>T<br>T<br>T	(Jeleń and Wąsowicz, 1998)<br>(Josselin *et al.*, 2021)<br>(Sun *et al.*, 2016)<br>(Zeringue, Bhatnagar and Cleveland, 1993)	[41]<br>[36]<br>[40]<br>[45]	/<br>PDA<br>CDA, CSA, CDL, MEA<br>Adye and Mateles liquid medium
alpha-farnesene	502-61-4	Terpene	T	(Sun *et al.*, 2016)	[40]	CDA, CSA, CDL, CMA
3,7-dimethylocta-1,3,7-triene	502-99-8	Alkene	T	(Sun *et al.*, 2016)	[40]	CDA,CDL, MEA

3-methylbutanoic acid	503-74-2	Acid	T	(De Lucca *et al.*, 2010)	[34]	Cracked maize
hex-2-enal	505-57-7	Aldehyde	T	(De Lucca *et al.*, 2012)	[35]	Non-sterile cracked maize
(E)-9-octadecen-1-ol	506-42-3	Alcohol	T	(De Lucca *et al.*, 2012)	[35]	Non-sterile cracked maize
2-methylbut-2-ene	513-35-9	Alkene	T	(De Lucca *et al.*, 2012)	[35]	Non-sterile cracked maize
butan-2,3-diol	513-85-9	Alcohol	NT/T<br>NT/T	(De Lucca *et al.*, 2010)<br>(Josselin *et al.*, 2021)	[34]<br>[36]	Cracked maize<br>PDA
3-hydroxybutan-2-one	513-86-0	Ketone	NT/T<br>NT	(Josselin *et al.*, 2021)<br>(Sun *et al.*, 2014)	[36]<br>[39]	PDA<br>Maize media
beta-elemene	515-13-9	Terpene	T<br>T	(Josselin *et al.*, 2021)<br>(Sun *et al.*, 2016)	[36]<br>[40]	PDA<br>CDA, CSA, CDL, MEA, CMA
beta-cadinene	523-47-7	Terpene	T<br>T	(Josselin *et al.*, 2021)<br>(Sun *et al.*, 2016)	[36]<br>[40]	PDA<br>CDA, CSA, CDL, MEA
cadinene	523-47-7	Terpene	U<br>T	(Jeleń and Wąsowicz, 1998)<br>(Zeringue, Bhatnagar and Cleveland, 1993)	[41]<br>[45]	/<br>Adye and Mateles liquid medium
3,4,5-trimethylphenol	527-54-8	Alcohol	T	(De Lucca *et al.*, 2012)	[35]	Non-sterile cracked maize
9-butyldocosane	5282-14-9	Alkane	T	(De Lucca *et al.*, 2010)	[34]	Cracked maize
2-methylfuran	534-22-5	Furan	NT/T<br>T<br>U<br>T<br>T<br>T	(De Lucca *et al.*, 2010)<br>(De Lucca *et al.*, 2012)<br>(Jeleń and Wąsowicz, 1998)<br>(Josselin *et al.*, 2021)<br>(Sun *et al.*, 2014)<br>(Sun *et al.*, 2016)	[34]<br>[35]<br>[41]<br>[33]<br>[39]<br>[40]	Cracked maize<br>Sterile cracked maize<br>/<br>PDA<br>Maize media<br>CDA, CSA, CDL, MEA, CMA
2-methylnon-3-ene	53966-53-3	Alkene	T	(De Lucca *et al.*, 2012)	[35]	Non-sterile cracked maize
2, 2, 4-trimethylpentane	540-84-1	Alkane	T	(Spraker *et al.*, 2014)	[44]	Glucose minimal medium (GMM)
2,6-dimethylheptadecane	54105-67-8	Alkane	T	(De Lucca *et al.*, 2012)	[35]	Non-sterile cracked maize
3-methylbutanamide	541-46-8	Amide	T	(De Lucca *et al.*, 2012)	[35]	Non-sterile cracked maize
epi-bicyclosequiphellandrene	54274-73-5	Terpene	T<br>U<br>T<br>T	(De Lucca *et al.*, 2012)<br>(Jeleń and Wąsowicz, 1998)<br>(Josselin *et al.*, 2021)<br>(Zeringue, Bhatnagar and Cleveland, 1993)	[35]<br>[41]<br>[36]<br>[45]	Non-sterile cracked maize<br>/<br>PDA<br>Adye and Mateles liquid medium
heptan-2-ol	543-49-7	Alcohol	T	(Sun *et al.*, 2014)	[39]	Maize media
1-chloropentane	543-59-9	Halogen	T	(De Lucca *et al.*, 2010)	[34]	Cracked maize
hexadecane	544-76-3	Alkane	NT+NT/T<br>T	(De Lucca *et al.*, 2010)<br>(De Lucca *et al.*, 2012)	[34]<br>[35]	PDA + Cracked maize<br>Non-sterile cracked maize
dotriacotane	544-85-4	Alkane	T	(De Lucca *et al.*, 2010)	[34]	Cracked maize
decyl formate	5451-52-5	Ester	T	(De Lucca *et al.*, 2012)	[35]	Non-sterile cracked maize
beta-cedrene	546-28-1	Terpene	U	(Polizzi *et al.*, 2012)	[38]	Wallpaper
10-methyleicosane	54833-23-7	Alkane	T	(De Lucca *et al.*, 2010)	[34]	Cracked maize
6-propyltridecane	55045-10-8	Alkane	T	(De Lucca *et al.*, 2010)	[34]	Cracked maize
viridiflorol	552-02-3	Terpene	T	(Josselin *et al.*, 2021)	[36]	PDA
5,14-dibutyloctadecane	55282-13-8	Alkane	T	(De Lucca *et al.*, 2010)	[34]	Cracked maize
3-ethyltetracosane	55282-17-2	Alkane	T	(De Lucca *et al.*, 2010)	[34]	Cracked maize
11-decylheneicosane	55320-06-4	Alkane	T	(De Lucca *et al.*, 2012)	[35]	Non-sterile cracked maize
3,7,7-trimethylbicyclo4.1.0]hept-2-ene	554-61-0	Terpene	T<br>T	(De Lucca *et al.*, 2010)<br>(De Lucca *et al.*, 2012)	[34]<br>[35]	Cracked maize<br>Non-sterile cracked maize
methyl 3-methylbutyrate	556-24-1	Ester	NT/T	(Sun *et al.*, 2014)	[39]	Maize media
2,3,3-trimethylpentane	560-21-4	Alkane	NT/T	(Sun *et al.*, 2014)	[39]	Maize media

o-(2-methylpropyl)hydroxylamine	5618-62-2	Amine	T	(Spraker *et al.*, 2014)	[44]	Glucose minimal medium (GMM)
3-methylbut-1-ene	563-45-1	Alkene	T	(De Lucca *et al.*, 2012)	[35]	Sterile cracked maize
2-methylbut-1-ene	563-46-2	Alkene	T	(De Lucca *et al.*, 2012)	[35]	Non-sterile cracked maize
3-methylpentan-2-one	565-61-7	Ketone	T	(De Lucca *et al.*, 2012)	[35]	Non-sterile cracked maize
beta-panasinsene	56684-97-0	Terpene	T	(Sun *et al.*, 2016)	[40]	CDA, CSA, CDL, MEA
2,3-dimethylhexane	584-94-1	Alkane	NT/T	(Sun *et al.*, 2014)	[39]	Maize media
2,4-dimethylhexane	589-43-5	Alkane	NT	(Sun *et al.*, 2014)	[39]	Maize media
octan-3-ol	589-98-0	Alcohol	U	(Jeleń and Wąsowicz, 1998)	[41]	/
3-methylbutanal	590-86-3	Aldehyde	NT/T<br>NT/T	(Josselin *et al.*, 2021)<br>(Sun *et al.*, 2014)	[36]<br>[39]	PDA<br>Maize media
hexan-2-one	591-78-6	Ketone	NT/T	(De Lucca *et al.*, 2012)	[35]	Non-sterile cracked maize
penta-1,4-diene	591-93-5	Alkene	T<br>T	(De Lucca *et al.*, 2012)<br>(Sun *et al.*, 2016)	[35]<br>[40]	Sterile cracked maize<br>CSA, CDL, MEA,CMA
penta-1,2-diene	591-95-7	Alkene	T	(De Lucca *et al.*, 2012)	[35]	Sterile cracked maize
2,5-dimethylhexane	592-13-2	Alkane	NT	(Sun *et al.*, 2014)	[39]	Maize media
hept-1-ene*	592-76-7	Alkene	T	(De Lucca *et al.*, 2012)	[35]	Non-sterile cracked maize
heptacosane	593-49-7	Alkane	T	(Sun *et al.*, 2016)	[40]	CDA, CDL, MEA, CMA
2,2,3,3-tetramethylbutane	594-82-1	Alkane	NT/T	(Sun *et al.*, 2014)	[39]	Maize media
palustrol	5986-49-2	Terpene	T	(Josselin *et al.*, 2021)	[36]	PDA
3-methylbutan-2-amine	598-74-3	Amine	NT	(De Lucca *et al.*, 2010)	[34]	PDA
phenylethyl alcohol	60-12-8	Alcohol	T	(De Lucca *et al.*, 2010)	[34]	Cracked maize
2,2’-dimethylbiphenyl	605-39-0	Alkene	T	(De Lucca *et al.*, 2010)	[34]	Cracked maize
(Z)-don-2-enal	60784-31-8	Aldehyde	T	(De Lucca *et al.*, 2012)	[35]	Non-sterile cracked maize
4,6-dimethyldodecane	61141-72-8	Alkane	T	(Josselin *et al.*, 2021)	[36]	PDA
4-methyldodecane	6117-97-1	Alkane	T	(De Lucca *et al.*, 2012)	[35]	Non-sterile cracked maize
4-methylheptan-3-one	6137-11-7	Ketone	T	(De Lucca *et al.*, 2012)	[35]	Non-sterile cracked maize
4-methyl-2-propyl furan	6148-37-4	Furan	T	(De Lucca *et al.*, 2012)	[35]	Non-sterile cracked maize
1-penten-3-ol	616-25-1	Alcohol	U	(Jeleń and Wąsowicz, 1998)	[41]	/
3-methylthiophene	616-44-4	Other	T	(De Lucca *et al.*, 2010)	[34]	Cracked maize
7-methylpentadecane	6165-40-8	Alkane	T	(De Lucca *et al.*, 2012)	[35]	Non-sterile cracked maize
3-phenoxypropan-1-ol	6180-61-6	Alcohol	T	(De Lucca *et al.*, 2010)	[34]	PDA + Cracked maize
2,3,6-trimethyloctane	62016-33-5	Alkane	T	(De Lucca *et al.*, 2010)	[34]	Cracked maize
2,4,6-trimethyldecane	62108-27-4	Alkane	T<br>T	(De Lucca *et al.*, 2010)<br>(De Lucca *et al.*, 2012)	[34]<br>[35]	Cracked maize<br>Non-sterile cracked maize
1-ethyl-4-methylbenzene	622-96-8	Alkene	NT	(De Lucca *et al.*, 2012)	[35]	Non-sterile cracked maize
benzenamine	62-53-3	Amine	T	(De Lucca *et al.*, 2010)	[34]	Cracked maize
2,5-dimethylfuran	625-86-5	Furan	T<br>NT/T	(De Lucca *et al.*, 2010)<br>(Josselin *et al.*, 2021)	[34]<br>[36]	Cracked maize<br>PDA
pent-2-yn-1-ol	6261-22-9	Alcohol	T	(De Lucca *et al.*, 2012)	[35]	Non-sterile cracked maize
1-methoxy-3-methylbutane	626-91-5	Alkane	T	(De Lucca *et al.*, 2012)	[35]	Non-sterile cracked maize
hexan-2-ol	626-93-7	Alcohol	T	(De Lucca *et al.*, 2012)	[35]	Sterile cracked maize
pent-1-yne	627-19-0	Alkyne	T	(De Lucca *et al.*, 2012)	[35]	Non-sterile cracked maize
1,4-cyclohexadiene	628-41-1	Alkene	T	(De Lucca *et al.*, 2012)	[35]	Non-sterile cracked maize
nonan-2-ol	628-99-9	Alcohol	T	(De Lucca *et al.*, 2012)	[35]	Non-sterile cracked maize
tridecane	629-50-5	Alkane	NT/T<br>T	(De Lucca *et al.*, 2010)<br>(De Lucca *et al.*, 2012)	[34]<br>[35]	Cracked maize<br>Non-sterile cracked maize

tetradecane	629-59-4	Alkane	T	(De Lucca *et al.*, 2010)	[34]	Cracked maize
pentadecane	629-62-9	Alkane	NT/T<br>T	(De Lucca *et al.*, 2010)<br>(De Lucca *et al.*, 2012)	[34]<br>[35]	Cracked maize<br>Sterile cracked maize
heptadecane	629-78-7	Alkane	T<br>T<br>T	(De Lucca *et al.*, 2010)<br>(De Lucca *et al.*, 2012)<br>(Josselin *et al.*, 2021)	[34]<br>[35]<br>[36]	PDA<br>Non-sterile cracked maize<br>PDA
nonadecane	629-92-5	Alkane	T<br>T/NT	(De Lucca *et al.*, 2010)<br>(De Lucca *et al.*, 2012)	[34]<br>[35]	Cracked maize<br>Non-sterile cracked maize
heneicosane	629-94-7	Alkane	NT/T	(De Lucca *et al.*, 2010)	[34]	Cracked maize
eicosan-1-ol	629-96-9	Alcohol	T	(De Lucca *et al.*, 2010)	[34]	Cracked maize
docosane	629-97-0	Alkane	NT	(De Lucca *et al.*, 2010)	[34]	Cracked maize
octacosane	630-02-4	Alkane	T<br>T	(De Lucca *et al.*, 2010)<br>(De Lucca *et al.*, 2012)	[34]<br>[35]	Cracked maize, Sterile<br>Non sterile cracked maize
nonacosane	630-03-5	Alkane	T<br>T	(De Lucca *et al.*, 2010)<br>(De Lucca *et al.*, 2012)	[34]<br>[35]	Cracked maize<br>Sterile cracked maize
isopropyl butanoate	638-11-9	Ester	T	(De Lucca *et al.*, 2010)	[34]	Cracked maize
2,6,10,14-tetramethylhexadecane	638-36-8	Alkane	T	(De Lucca *et al.*, 2012)	[35]	Non-sterile cracked maize
ethanol	64-17-5	Alcohol	T<br>T<br>NT/T<br>T	(De Lucca *et al.*, 2010)<br>(De Lucca *et al.*, 2012)<br>(Josselin *et al.*, 2021)<br>(Sun *et al.*, 2016)	[34]<br>[35]<br>[36]<br>[40]	Cracked maize<br>Sterile cracked maize<br>PDA<br>CDA, CSA, CDL, MEA, CMA
3-methylhexadecane	6418-43-5	Alkane	T<br>T	(De Lucca *et al.*, 2010)<br>(De Lucca *et al.*, 2012)	[34]<br>[35]	Cracked maize<br>Non-sterile cracked maize
3-methyleicosane	6418-46-8	Alkane	T	(De Lucca *et al.*, 2012)	[35]	Sterile cracked maize
acetic acid	64-19-7	Acid	NT/T<br>NT/T<br>T*	(De Lucca *et al.*, 2010)<br>(Josselin *et al.*, 2021)<br>(Sun *et al.*, 2014)	[34]<br>[36]<br>[39]	Cracked maize<br>PDA<br>Maize media
(Z)-5-octen-1-ol	64275-73-6	Alcohol	T	(De Lucca *et al.*, 2012)	[35]	Non-sterile cracked maize
2-methylbiphenyl	643-58-3	Alkene	T	(De Lucca *et al.*, 2010)	[34]	Cracked maize
3-methyl-1,1''-biphenyl	643-93-6	Alkane	NT/T	(De Lucca *et al.*, 2010)	[34]	Cracked maize
alpha-curcumene	644-30-4	Terpene	U	(Polizzi *et al.*, 2012)	[38]	wallpaper
(E)-pent-2-ene	646-04-8	Alkene	T<br>NT/T	(De Lucca *et al.*, 2010)<br>(De Lucca *et al.*, 2012)	[34]<br>[35]	Non-sterile cracked maize<br>Sterile cracked maize
tetracosane	646-31-1	Alkane	T<br>T	(De Lucca *et al.*, 2010)<br>(De Lucca *et al.*, 2012)	[34]<br>[35]	Cracked maize<br>Sterile cracked maize
alpha-isocomene	65372-78-3	Terpene	T	(Josselin *et al.*, 2021)	[36]	PDA
benzoic acid	65-85-0	Acid	NT<br>T	(De Lucca *et al.*, 2010)<br>(De Lucca *et al.*, 2012)	[34]<br>[35]	PDA<br>Non-sterile cracked maize
hexanal	66-25-1	Aldehyde	NT<br>T	(Sun *et al.*, 2014)<br>(De Lucca *et al.*, 2010)	[39]<br>[34]	Maize media<br>Cracked maize
(4E,6E)-2,6-dimethylocta-2,4,6-triene	673-84-7	Alkene	T	(De Lucca *et al.*, 2012)	[35]	Non-sterile cracked maize
2,6-dimethylocta-5,7-dien-4-one	6752-80-3	Ketone	NT	(De Lucca *et al.*, 2010)	[34]	PDA
propan-2-ol	67-63-0	Alcohol	NT<br>T	(Josselin *et al.*, 2021)<br>(Sun *et al.*, 2016)	[36]<br>[40]	PDA<br>CDA, CSA, MEA, CMA
acetone	67-64-1	Ketone	NT/T<br>T	(Sun *et al.*, 2014)<br>(Sun *et al.*, 2016)	[39]<br>[40]	Maize media<br>CSA, CDL, MEA, CMA
bicyclogermacrene	67650-90-2	Terpene	T	(De Lucca *et al.*, 2012)	[35]	Non-sterile cracked maize

trichloromethane	67-66-3	Halogen	NT/T<br>T	(Josselin *et al.*, 2021)<br>(Spraker *et al.*, 2014)	[36]<br>[44]	PDA<br>Glucose minimal medium (GMM)
1,1,2,3-tetramethylcyclohexane	6783-92-2	Alkane	T	(De Lucca *et al.*, 2012)	[35]	Non-sterile cracked maize
aristolene	6831-16-9	Terpene	U<br>T<br>T	(Jeleń and Wąsowicz, 1998)<br>(Sun *et al.*, 2016)<br>(Zeringue, Bhatnagar and Cleveland, 1993)	[41]<br>[40]<br>[45]	/<br>CDA, CSA, CDL, MEA, CMA<br>Adye and Mateles liquid medium
dipropan-2-yl hexanedioate	6938-94-9	Acid	T	(De Lucca *et al.*, 2010)	[34]	Cracked maize
bicyclo[4.2.0]octa-1,3,5-triene	694-87-1	Alkene	T<br>NT	(De Lucca *et al.*, 2010)<br>(De Lucca *et al.*, 2012)	[34]<br>[35]	Cracked maize<br>Sterile cracked maize
pentatriacont-17-ene	6971-40-0	Alkene	T	(De Lucca *et al.*, 2010)	[34]	Cracked maize
propan-1-ol	71-23-8	Alcohol	NT/T<br>NT/T	(Spraker *et al.*, 2014)<br>(Josselin *et al.*, 2021)	[45]<br>[36]	Maize media<br>PDA
butan-1-ol	71-36-3	Alcohol	T<br>T*	(Spraker *et al.*, 2014)<br>(Josselin *et al.*, 2021)	[44]<br>[36]	Glucose minimal medium (GMM)<br>PDA
pentan-1-ol	71-41-0	Alcohol	NT/T	(Spraker *et al.*, 2014)	[44]	Maize media
benzene	71-43-2	Alkene	T	(De Lucca *et al.*, 2012)	[35]	Non-sterile cracked maize
2,3-dimethyloctane	7146-60-3	Alkane	T	(De Lucca *et al.*, 2012)	[35]	Non-sterile cracked maize
di-epi-1,10-cubenol	73365-77-2	Terpene	T*	(Josselin *et al.*, 2021)	[36]	PDA
ethyl 2-methylbutyrate	7452-79-1	Ester	NT<br>NT/T*<br>NT/T	(De Lucca *et al.*, 2010)<br>(Josselin *et al.*, 2021)<br>(Sun *et al.*, 2014)	[34]<br>[36]<br>[39]	PDA<br>PDA<br>Maize media
nonyl-cyclopropane	74663-85-7	Alkane	T	(Josselin *et al.*, 2021)	[36]	PDA
1-ethyl-2-heptylcyclopropane	74663-86-8	Alkane	T	(De Lucca *et al.*, 2012)	[35]	Non-sterile cracked maize
acetaldehyde	75-07-0	Aldehyde	T	(Josselin *et al.*, 2021)	[36]	PDA
2,6-dimethyloctadecane	75163-97-2	Alkane	T	(De Lucca *et al.*, 2012)	[35]	Non-sterile cracked maize
dimethyl sulfide	75-18-3	Other	T<br>U<br>T	(De Lucca *et al.*, 2010)<br>(Gao *et al.*, 2002)<br>(Sun *et al.*, 2014)	[34]<br>[42]<br>[39]	Cracked maize<br>Gypsum board<br>Maize media
cyclopropane	75-19-4	Alkane	U	(De Lucca *et al.*, 2012)	[35]	Non-sterile cracked maize
nitromethane	75-52-5	Halogen	U	(Jeleń and Wąsowicz, 1998)	[41]	/
3-methylbut-3-en-1-ol	763-32-6	Alcohol	T	(De Lucca *et al.*, 2012)	[35]	Non-sterile cracked maize
2,2-dimethylbutane-1,3-diol	76-35-7	Alcohol	T	(De Lucca *et al.*, 2012)	[35]	Non-sterile cracked maize
5-methylhexa-1,4-diene	763-88-2	Alkene	T	(De Lucca *et al.*, 2012)	[35]	Non-sterile cracked maize
bicyclo2.2.0]hexa-2,5-diene	7641-77-2	Alkene	T*	(Josselin *et al.*, 2021)	[36]	PDA
hex-2-yne	764-35-2	Alkyne	T	(De Lucca *et al.*, 2012)	[35]	Non-sterile cracked maize
1-phenoxypropan-2-ol	770-35-4	Alcohol	NT	(Jeleń and Wąsowicz, 1998)	[41]	PDA
4-ethenyl-2-methoxyphenol	7786-61-0	Alcohol	T	(De Lucca *et al.*, 2010)	[34]	Cracked maize
alpha dehydro-ar-himachalene	78204-62-3	Terpene	T	(Josselin *et al.*, 2021)	[36]	PDA
2-methylpropan-1-ol	78-83-1	Alcohol	NT/T<br>T<br>U<br>U<br>T<br>NT/T	(De Lucca *et al.*, 2010)<br>(Gao *et al.*, 2002)<br>(Jeleń and Wąsowicz, 1998)<br>(Josselin *et al.*, 2021)<br>(Spraker *et al.*, 2014)<br>(Zeringue, Bhatnagar and Cleveland, 1993)*	[34]<br>[42]<br>[41]<br>[36]<br>[44]<br>[45]	Maize media<br>PDA + Cracked maize<br>Gypsum board+Malt extract agar<br>/<br>PDA<br>/<br>MEA + Gympsum board
2-methylpropanal	78-84-2	Aldehyde	NT	(Sun *et al.*, 2014)	[39]	Maize media
2-methylpropenal	78-85-3	Aldehyde	T	(De Lucca *et al.*, 2012)	[35]	Sterile + non-sterile cracked maize

butan-2-one	78-93-3	Ketone	T*	(Josselin *et al.*, 2021)	[36]	PDA
2,3-dimethylbutane	79-29-8	Alkane	T	(De Lucca *et al.*, 2010)	[34]	Cracked maize
2-methylpropanoic acid	79-31-2	Acid	T<br>T*<br>NT/T	(De Lucca *et al.*, 2010)<br>(Josselin *et al.*, 2021)<br>(Sun *et al.*, 2014)	[34]<br>[36]<br>[39]	Cracked maize<br>PDA<br>Maize media
terpineol	8006-39-1	Terpene	U	(Gao *et al.*, 2002) *	[42]	Gypsum board
alpha-pinene	80-56-8	Terpene	T<br>NT	(De Lucca *et al.*, 2012)<br>(Sun *et al.*, 2014)	[35]<br>[39]	Non-sterile cracked maize<br>Maize media
1,5-hexadien-3-yne	821-08-9	Alkyne	NT	(De Lucca *et al.*, 2012)	[35]	Non-sterile cracked maize
nonan-2-one	821-55-6	Ketone	NT/T	(Sun *et al.*, 2014)	[39]	Maize media
(E)-2-octenoic acid	871-67-6	Acid	T	(De Lucca *et al.*, 2012)	[35]	Non-sterile cracked maize
(E)-caryophyllene	87-44-5	Terpene	T<br>T<br>T	(Jeleń and Wąsowicz, 1998)<br>(Josselin *et al.*, 2021)<br>(Zeringue, Bhatnagar and Cleveland, 1993)	[41]<br>[36]<br>[45]	Adye and Mateles liquid medium<br>PDA<br>/
isobazzanene	88661-59-0	Terpene	U	(Polizzi *et al.*, 2012)	[38]	Wallpaper
2,3,4-trimethylhexane	921-47-1	Alkane	NT/T	(Sun *et al.*, 2014)	[39]	Maize media
biphenyl	92-52-4	Alkane	NT	(De Lucca *et al.*, 2010)	[34]	PDA
hex-3-yne	928-49-4	Alkyne	T	(De Lucca *et al.*, 2012)	[35]	Non-sterile cracked maize
6-methylheptan-2-one	928-68-7	Ketone	U	(Polizzi *et al.*, 2012)*	[38]	Malt extract agar
1,3,6-octatriene	929-20-4	Alkene	T	(De Lucca *et al.*, 2012)	[35]	Non-sterile cracked maize
1-methoxyoctane	929-56-6	Alkane	T	(De Lucca *et al.*, 2012)	[35]	Non-sterile cracked maize
1-ethenoxyoctadecane	930-02-9	Alkane	T	(De Lucca *et al.*, 2012)	[35]	Non-sterile cracked maize
3-methylfuran	930-27-8	Furan	U<br>NT	(Jeleń and Wąsowicz, 1998)<br>(De Lucca *et al.*, 2012)	[41]<br>[35]	/<br>Non-sterile cracked maize
butylcyclopropane	930-57-4	Alkane	T	(De Lucca *et al.*, 2012)	[35]	Non-sterile cracked maize
4-ethyl-1,2-dimethylbenzene	934-80-5	Alkene	T	(De Lucca *et al.*, 2012)	[35]	Non-sterile cracked maize
dimethylbenzene	95-47-6	Alkene	U	(Jeleń and Wąsowicz, 1998)	[41]	/
1,2-dimethylbenzene	95-47-6	Alkene	T	(De Lucca *et al.*, 2012)	[35]	Non-sterile cracked maize
3,4-dimethylphenol	95-65-8	Alcohol	T	(De Lucca *et al.*, 2012)	[35]	Non-sterile cracked maize
isoledene	95910-36-4	Terperne	T	(Sun *et al.*, 2016)	[40]	CSA, CDL, MEA, CMA
3-methylpentane	96-14-0	Alkane	T	(De Lucca *et al.*, 2012)	[35]	Non-sterile cracked maize
2-methylbutanal	96-17-3	Aldehyde	NT/T<br>NT/T	(Sun *et al.*, 2014)<br>(Josselin *et al.*, 2021)	[39]<br>[36]	Maize media<br>PDA
butyrolactone	96-48-0	Other	T	(De Lucca *et al.*, 2010)	[34]	Cracked maize
ethyl 2-methylpropionate	97-62-1	Ester	T<br>NT/T<br>NT/T	(De Lucca *et al.*, 2012)<br>(Sun *et al.*, 2014)<br>(Josselin *et al.*, 2021)	[35]<br>[39]<br>[36]	Non-sterile cracked maize<br>Maize media<br>PDA
furfural	98-01-1	Aldehyde	T<br>T	(De Lucca *et al.*, 2010)<br>(De Lucca *et al.*, 2012)	[34]<br>[35]	Crackedv+Sterile cracked maize<br>Non-sterile cracked maize
acetophenone	98-86-2	Ketone	NT/T	(De Lucca *et al.*, 2010)	[34]	Cracked maize
(Z)-(1s,3s,6r)-4-carene	Not available	Alkene	T	(De Lucca *et al.*, 2012)	[35]	Non-sterile cracked maize
1-(3-propoxyphenyl)propan-2-amine	Not available	Amine	T	(De Lucca *et al.*, 2012)	[35]	Sterile cracked maize
1-(4-aminophenyl-3-phenyl-2-propen-1-one	Not available	Ketone	NT	(De Lucca *et al.*, 2010)	[34]	PDA
1-butylhexene	Not available	Alkene	T	(De Lucca *et al.*, 2012)	[35]	Non-sterile cracked maize
1-ethyl-methylbenzene	Not available	Alkene	NT	(De Lucca *et al.*, 2012)	[35]	Non-sterile cracked maize
1-iodo-2-methylnonane	Not available	Halogen	T	(De Lucca *et al.*, 2010)	[34]	Cracked maize

1-methoxypentadecane	Not available	Alkane	T	(De Lucca *et al.*, 2012)	[35]	Sterile cracked maize
2-butanoic acid	Not available	Acid	T	(De Lucca *et al.*, 2010)	[34]	Cracked maize
3-fluoro-a, 5-dihydroxy-N-methyl-benzeneethanamine	Not available	Amine	T	(Spraker *et al.*, 2014)	[44]	Glucose minimal medium (GMM)
3-methyl-1-butyl acetate	Not available	Ester	T	(De Lucca *et al.*, 2010)	[34]	Cracked maize
4-(1-methylethyl)phenol	Not available	Alcohol	T	(De Lucca *et al.*, 2012)	[35]	Non-sterile cracked maize
alpha-chamipinene	Not available	Terpene	T	(Polizzi *et al.*, 2012)	[38]	Wallpaper
benzaldehyde, 4-(methyoxyethyl) acetate	Not available	Ester	T	(De Lucca *et al.*, 2012)	[35]	Non-sterile cracked maize
beta-germacrene	Not available	Terpene	T	(Sun *et al.*, 2016)	[40]	CDA, CSA, CDL, MEA
esorubin hydrochloride*	Not available	Other	T	(De Lucca *et al.*, 2012)	[35]	Non-sterile cracked maize
hexaosan-1-ol	Not available	Alcohol	T	(De Lucca *et al.*, 2012)	[35]	Non-sterile cracked maize
methoxyphenyl oxime	Not available	Other	NT/T	(De Lucca *et al.*, 2010)	[34]	Cracked maize
n-(4-phenylazo)phenyl-2-phenylcyclopropionamide	Not available	Ester	T	(De Lucca *et al.*, 2010)	[34]	Cracked maize
4-methyl-1-91-methylethyl)bicycle3.1.0]hexan-3-ol	Not available	Alcohol	T	(De Lucca *et al.*, 2010)	[34]	Cracked maize
tetracyclo[3.3.1.1(1,8).0(2,4)]decane	Not available	Alkane	T	(De Lucca *et al.*, 2012)	[35]	Non-sterile cracked maize
6-isopropenyl-1,2,3,4-tetramethyl-1,4-cyclohexadiene	Not available	Alkene	NT	(De Lucca *et al.*, 2012)	[35]	Non-sterile cracked maize
(E,Z)-1,2-diethylidenecyclopentane	Not available	Alkane	NT	(Josselin *et al.*, 2021)	[36]	PDA
2-(3,3-diphenyl-propylamino)ethanol	Not available	Alcohol	T	(De Lucca *et al.*, 2012)	[35]	Sterile cracked maize
1,4-methanonaphtalene	Not available	Alkene	T	(De Lucca *et al.*, 2010)	[34]	Cracked maize
1-(2,5-dimethoxy-4-methylsulfonylphenyl)propan-2-amine	Not available	Amine	T	(Spraker *et al.*, 2014)	[44]	Glucose minimal medium (GMM)
(7a-isopropenyl-4,5-dimethyloctahydroinden-4-yl)methanol	Not available	Terpene	T*	(Josselin *et al.*, 2021)	[36]	PDA
pyrrolidine,1-8-(3-octyloxiranyl)-1-oxooctyl]-	Not available	Other	T	(De Lucca *et al.*, 2012)	[35]	Non-sterile cracked maize

(a) IUPAC or common name of the VOC (b) VOC case number (c) Chemical family of the VOC (d) Toxigenicity of the A. flavus strain: (T) toxigenic, (NT) non-toxigenic, (T*) natural mutant and (U) data unknown (e) The literature reference in relation to (f) the substrate type [No data available (/); Potato dextrose agar (PDA); Malt extract agar (MEA), Czapek solution agar (CSA) and corn meal agar (CMA) chemical defined agar (CDA) chemical defined liquid (CDL)]

References

[34] De Lucca *et al.* (2010) “Volatile profiles of toxigenic and non-toxigenic Aspergillus flavus using SPME for solid phase extraction,” *Annals of Agricultural and Environmental Medicine*, 17(2), pp. 301–308.

[35] De Lucca, A. J. *et al.* (2012) “Volatile profiles and aflatoxin production by toxigenic and non-toxigenic isolates of Aspergillus flavus grown on sterile and non-sterile cracked corn,” *Annals of Agricultural and Environmental Medicine*, 19(1), pp. 91–98.

[36] Josselin, L. *et al.* (2021) “Volatile organic compounds emitted by Aspergillus flavus strains producing or not aflatoxin B1,” *Toxins*, 13(10). doi: 10.3390/toxins13100705.

[37] Müller, A. *et al.* (2013) “Volatile profiles of fungi - Chemotyping of species and ecological functions,” *Fungal Genetics and Biology*, 54, pp. 25–33. doi: 10.1016/j.fgb.2013.02.005.

[38] Polizzi, V. *et al.* (2012) “Identification of volatile markers for indoor fungal growth and chemotaxonomic classification of Aspergillus species,” *Fungal Biology*. Elsevier Ltd, 116(9), pp. 941–953. doi: 10.1016/j.funbio.2012.06.001.

[39] Sun, D. *et al.* (2014) “Monitoring MVOC Profiles over Time from Isolates of Aspergillus flavus Using SPME GC-MS,” *Journal of Agricultural Chemistry and Environment*, 03(02), pp. 48–63. doi: 10.4236/jacen.2014.32007.

[40] Sun, D. *et al.* (2016) “Effects of Growth Parameters on the Analysis of Aspergillus flavus Volatile Metabolites,” pp. 1–20. doi: 10.3390/separations3020013.

[41] Jeleń, H. and Wąsowicz, E. (1998) “Volatile fungal metabolites and their relation to the spoilage of agricultural commodities,” *Food Reviews International*, 14(4), pp. 391–426. doi: 10.1080/87559129809541170.

[42] Gao, P. *et al.* (2002) “Determination of unique microbial volatile organic compounds produced by five aspergillus species commonly found in problem buildings,” *American Industrial Hygiene Association Journal*, 63(2), pp. 135–140. doi: 10.1080/15428110208984696.

[43] Kamiński, E. *et al.* (1972) “Identification of the predominant volatile compounds produced by Aspergillus flavus.,” *Applied microbiology*, 24(5), pp. 721–6. Available at: http://www.pubmedcentral.nih.gov/articlerender.fcgi?artid=380652&tool=pmcentrez&rendertype=abstract.

[44] Spraker, J. E. *et al.* (2014) “A Volatile Relationship: Profiling an Inter-Kingdom Dialogue Between two Plant Pathogens, Ralstonia Solanacearum and Aspergillus Flavus,” *Journal of Chemical Ecology*, 40(5), pp. 502–513. doi: 10.1007/s10886-014-0432-2.

[45] Zeringue, H. J., Bhatnagar, D. and Cleveland, T. E. (1993) “C15H24 Volatile Compounds Unique to Aflatoxigenic Strains of Aspergillus flavus,” 59(7), pp. 2264–2270.
